# Cacao powder supplementation attenuates oxidative stress, cholinergic impairment, and apoptosis in d-galactose-induced aging rat brain

**DOI:** 10.1038/s41598-021-96800-y

**Published:** 2021-09-09

**Authors:** Hyoeun Yoo, Hyun-Sook Kim

**Affiliations:** Food and Nutrition, Graduate School of Life and Sciences, Sookmyung Women’s University, Seoul, Korea

**Keywords:** Biochemistry, Neuroscience, Biomarkers

## Abstract

Aging, a critical risk factor of several diseases, including neurodegenerative disorders, affects an ever-growing number of people. Cacao supplementation has been suggested to improve age-related neuronal deficits. Therefore, this study investigated the protective effects of raw cacao powder on oxidative stress-induced aging. Male Sprague–Dawley rats were divided into 4 groups: Control (C), d-galactose-induced aging (G), d-galactose injection with 10% (LC), and 16% (HC) cacao powder mixed diet. d-galactose (300 mg/3 mL/kg) was intraperitoneally injected into all but the control group for 12 weeks. Cacao supplemented diets were provided for 8 weeks. The levels of serum Malondialdehyde (MDA), Advanced Glycation End-products (AGEs), brain and liver MDA, the indicators of the d-galactose induced oxidative stress were significantly decreased in LC and HC but increased in G. The Acetylcholinesterase (AChE) activity of brain showed that the cholinergic impairment was significantly lower in LC, and HC than G. Furthermore, the expression levels of catalase (CAT), phospho-Akt/Akt, and procaspase-3 were significantly increased in LC and HC. In conclusion, cacao consumption attenuated the effects of oxidative stress, cholinergic impairment and apoptosis, indicating its potential in future clinical studies.

## Introduction

Aging is a critical risk factor of neurodegenerative disorders as it is a gradual process inducing dysfunction and degeneration of the nervous or immune system, which results in cognitive impairment^1,2^. Harman explained that aerobic respiration contributes to free radical production leading to the accumulation of oxidative damage, which may lead to aging and death^3^. The imbalance of production and removal of reactive oxygen species (ROS) induces oxidative stress^4^, and excessive ROS or deficient enzymatic antioxidants cause lipid peroxidation, DNA damage, and protein oxidation that may lead to dysfunction of the organism^5^. Additionally, malondialdehyde (MDA) and advanced glycation end-products (AGEs), which deteriorates with aging, were speculated to lead to all kinds of age-related disorders^6^. Several studies have reported the effect of apoptosis in aging and age-related disease; however, it is not clear whether aging suppresses or enhances apoptosis in vivo. The phosphatidylinositol 3-kinase (PI3K) and Akt (otherwise known as Protein Kinase B) pathway has been implicated as an anti-apoptotic pathway in many different cellular paradigms. Many studies have suggested that Akt regulates cell death regulation resulting in modulation of the degenerative diseases’ pathogenesis and cancer cells^3^. PI3K and its downstream Akt are suggested to be the vital enzymes in regulating cell growth, metabolism, and survival modulated by growth factors and neurotransmitters^4^. Activated PI3K induces the activation of Akt, and previous researches have explained that Akt activation may be therapeutic in neurodegenerative diseases^5^. Though the mechanism is not clear yet, several studies have investigated the neurodegenerative diseases related to modulation of PI3K/Akt signal dysregulation^5,6^. In addition, Caspase-3 has been shown to be a major effector of apoptosis in cells activated by oxidative stress and ROS^7^, and the inhibition of oxidative stress may suppress the apoptotic cell death^8^. Furthermore, it is known that the free radical scavenging enzymes such as SOD, CAT, and GPx exhibit antioxidative effects against oxidative stress^8^, and impart protection against aging induced by oxidative stress^9–11^. This could be due to the protective effects of decreasing oxidative stress against cell death, which inhibits the age-related changes in neuronal function, viability, and cognition^10^.

Many studies have investigated the concrete mechanism of aging; however, they are mutually conclusive. Several studies have reported d-galactose as a source of inducing animal senescence. They have shown increased oxidative stress, inflammatory damage, and apoptosis in the lungs, livers, kidneys, and brains of animals^12–15^. Therefore, d-galactose injected animals have been used as aging induced or organ injury models steadily.

The polyphenol-rich foods have gained an increasing research interest because of their potential antioxidative, neuroprotective and cognition enhancing effects^16–21^. Cacao contains a large content of polyphenols, including compounds such as (+)-catechin and (−)-epicatechin, as well as procyanidins (58%) that are effective ROS scavengers^21,22^. A previous study has shown that an LMN diet (a diet enriched in polyphenols and polyunsaturated fatty acids) composed of cocoa, nuts, vegetable oil rich in unsaponifiable fatty acids, and flours rich in soluble fibers exerts protective effects against neuronal loss caused by aging and delays the Aβ plaque formation^23^. Similarly, another research conducted with cocoa enriched diets provided evidence that cocoa can induce upregulation of mitogen-activated kinase (MAP) phosphatase MKP, prevent inflammatory responses in trigeminal ganglion neurons, and be an effective treatment of migraine^21^. (−)-Epicatechin, a flavanol in cacao, has shown preventive effect against stroke through Nrf2 activation and neuroprotective Heme Oxygenase-1(HO-1) increase^22^.

However, the mechanisms of the protective effects of cacao in aging models are not clear yet. To the best of our knowledge, this is the first study to investigate the protective effects of cacao powder in the d-galactose-induced aging model. The present study observed the d-galactose-induced oxidative stress and protective effects of cacao powder against oxidative stress, cholinergic impairment, and apoptosis.

## Results

### Body weight, organ coefficients, and food intake

**Figure 1:**
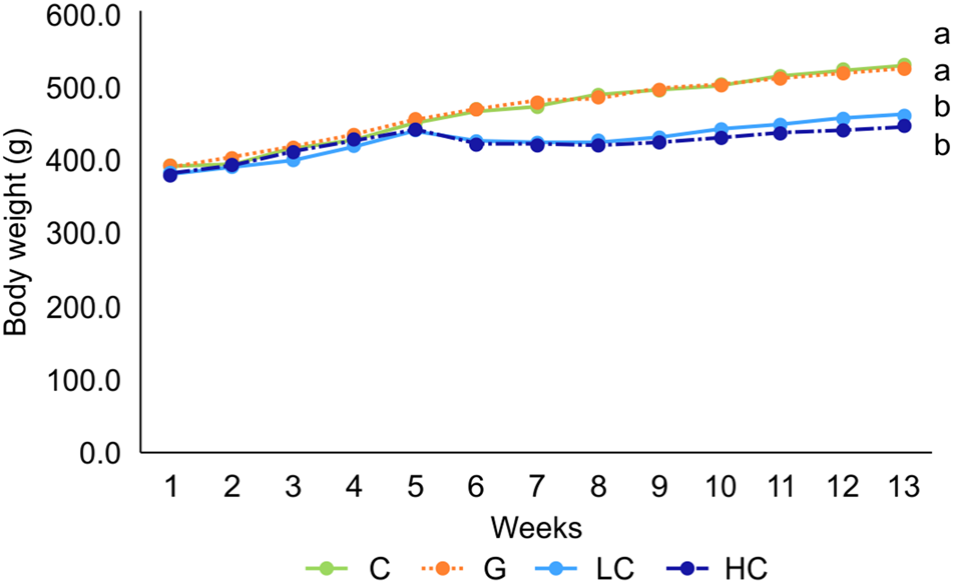
Body weight change observed during the experiment period in each group. Values represent mean ± SD, n = 8. ns: not significant; Means for a variable with different letters (a and b) within a column differ significantly (*p* < 0.05). C, control; G, d-galactose-induced aging; LC, d-galactose injection with 10% cacao powder mixed diet; HC, d-galactose injection with 16% cacao powder mixed diet.

**Figure 2:**
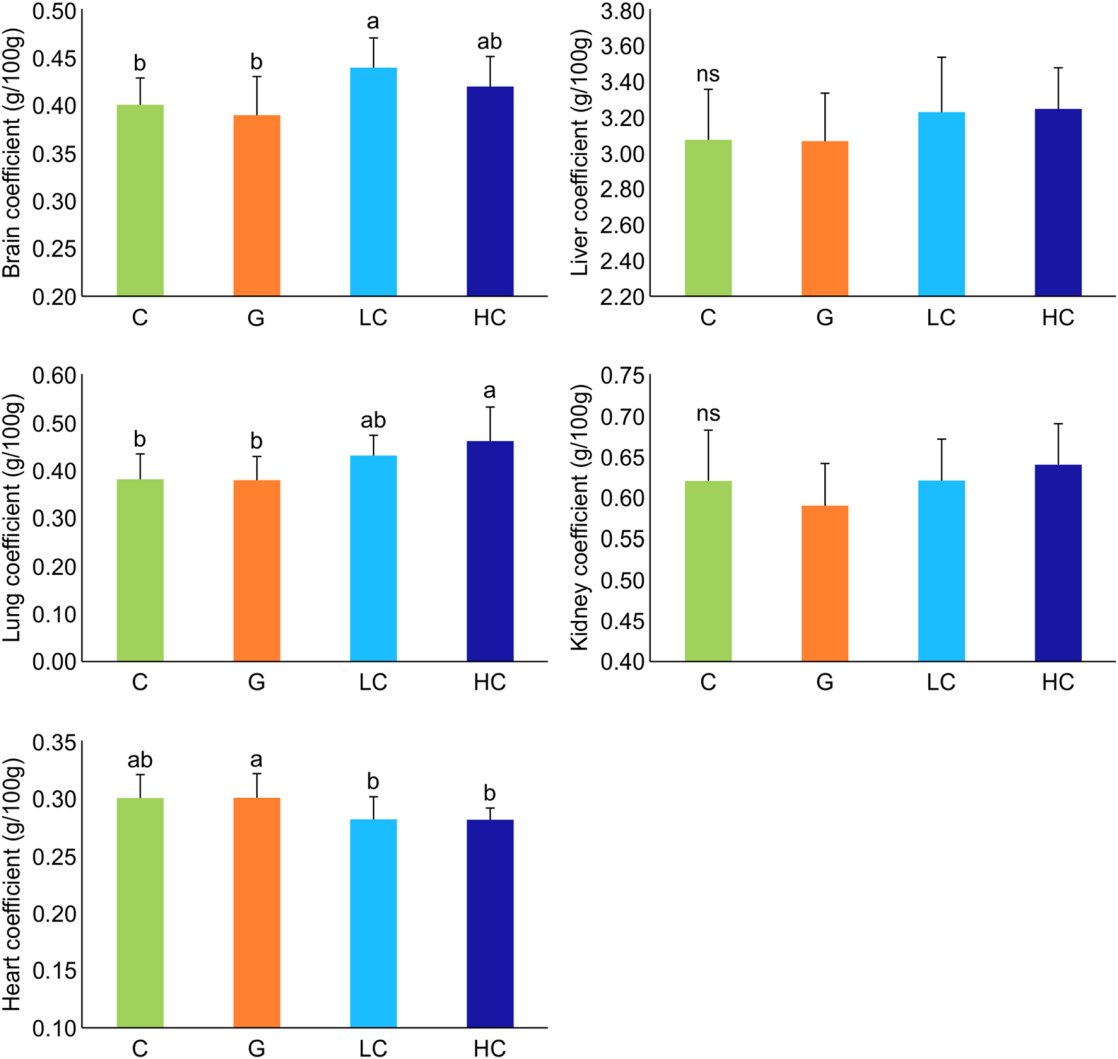
Organ coefficients of each group. Values represent mean ± SD, n = 8. ns: not significant; Means for a variable with different letters (a and b) within a column differ significantly (*p* < 0.05). C, control; G, d-galactose-induced aging; LC, d-galactose injection with 10% cacao powder mixed diet; HC, d-galactose injection with 16% cacao powder mixed diet.

The initial body weights showed no significant difference among the four groups. However, the final body weights and body weight gain of LC and HC groups were significantly lower compared to C and G groups (Table 1, Fig. 1). The amount of food intake was significantly lower in G, LC, and HC compared to the C group. Organ coefficients for the brains, livers, kidneys, hearts, and lungs are shown in Fig. 2. The coefficients of the brain, lung and heart were significantly higher in LC, HC, and G groups, respectively, compared to the other groups in each organ, while the coefficients of the liver and kidneys showed no significant differences among all groups.

**Table 1:** Initial body weight, final body weight and food intake of each group.

Group	Initial body weight (g)	Final body weight (g)	Body weight gain (g)	Food intake (g/day)
C	384.13 ± 16.75^ ns^	522.63 ± 36.40^a^	123.11 ± 56.74^a^	20.37 ± 1.41^a^
G	385.13 ± 15.41	521 ± 51.94^a^	135.88 ± 42.37^a^	19.59 ± 2.26^b^
LC	379.94 ± 15.36	458.75 ± 17.89^b^	70.06 ± 33.32^b^	19.14 ± 2.17^b^
HC	378.19 ± 12.86	444.38 ± 23.56^b^	66.19 ± 24.65^b^	19.37 ± 2.64^b^

Values represent mean ± SD, n = 8. ns: not significant; Means for a variable with different letters (a and b) within a column differ significantly (*p* < 0.05). C, control; G, d-galactose-induced aging; LC, d-galactose injection with 10% cacao powder mixed diet; HC, d-galactose injection with 16% cacao powder mixed diet.

### Oxidative stress protection with cacao in serum, liver, and brain

**Figure 3:**
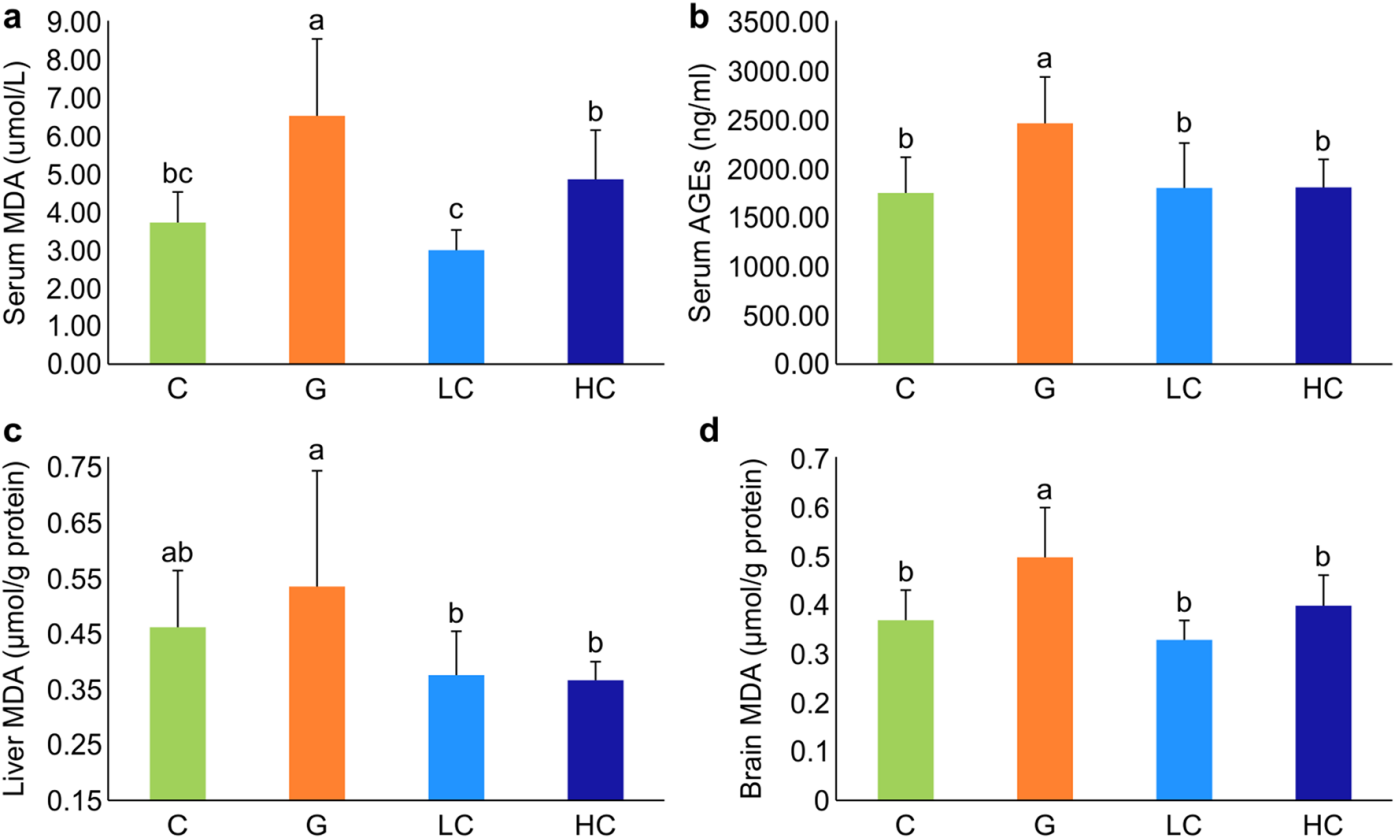
Quantitative analysis of MDA and AGEs generation induced by d-galactose in the presence of cacao. (**a**) Serum MDA level; (**b**) Serum AGEs level; (**c**) Liver MDA level; (**d**) Brain MDA level**.** Values represent mean ± SD, n = 8; Means for a variable with different letters (a, b, c) differ significantly (*p* < 0.05). C, control; G, d-galactose-induced aging; LC, d-galactose injection with 10% cacao powder mixed diet; HC, d-galactose injection with 16% cacao powder mixed diet.

Serum MDA and AGEs levels were significantly higher in the G group compared to the C group (Fig. 3a, b). The LC and HC groups showed significantly lower serum MDA levels than the G group (*p* < 0.001), whereas the AGEs level of the G group was significantly higher than the remaining three groups (*p* = 0.005). The liver and brain MDA levels were the highest in the G group and showed a significant decrease in the LC and HC groups compared to the G group (*p* = 0.034, < 0.001)(Fig. 3c, d).

### Amelioration of brain cholinergic system impairment with cacao

**Figure 4:**
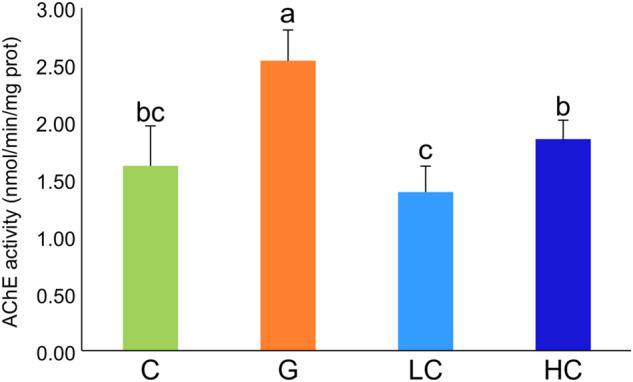
Brain AchE activity in each group. Values represent mean ± SD, n = 8. Means for a variable with different letters (a, b, c) differ significantly (*p* < 0.001). C, control; G, d-galactose-induced aging; LC, d-galactose injection with 10% cacao powder mixed diet; HC, d-galactose injection with 16% cacao powder mixed diet.

AChE activity was measured to assess cholinergic system impairment. The AChE activity in the brain tissue significantly increased in the G group as compared to the C group, whereas it was significantly decreased in both LC and HC groups as compared to the G group (*p* < 0.001) (Fig. 4).

### Effect of cacao on antioxidant enzymes in the brain

**Figure 5:**
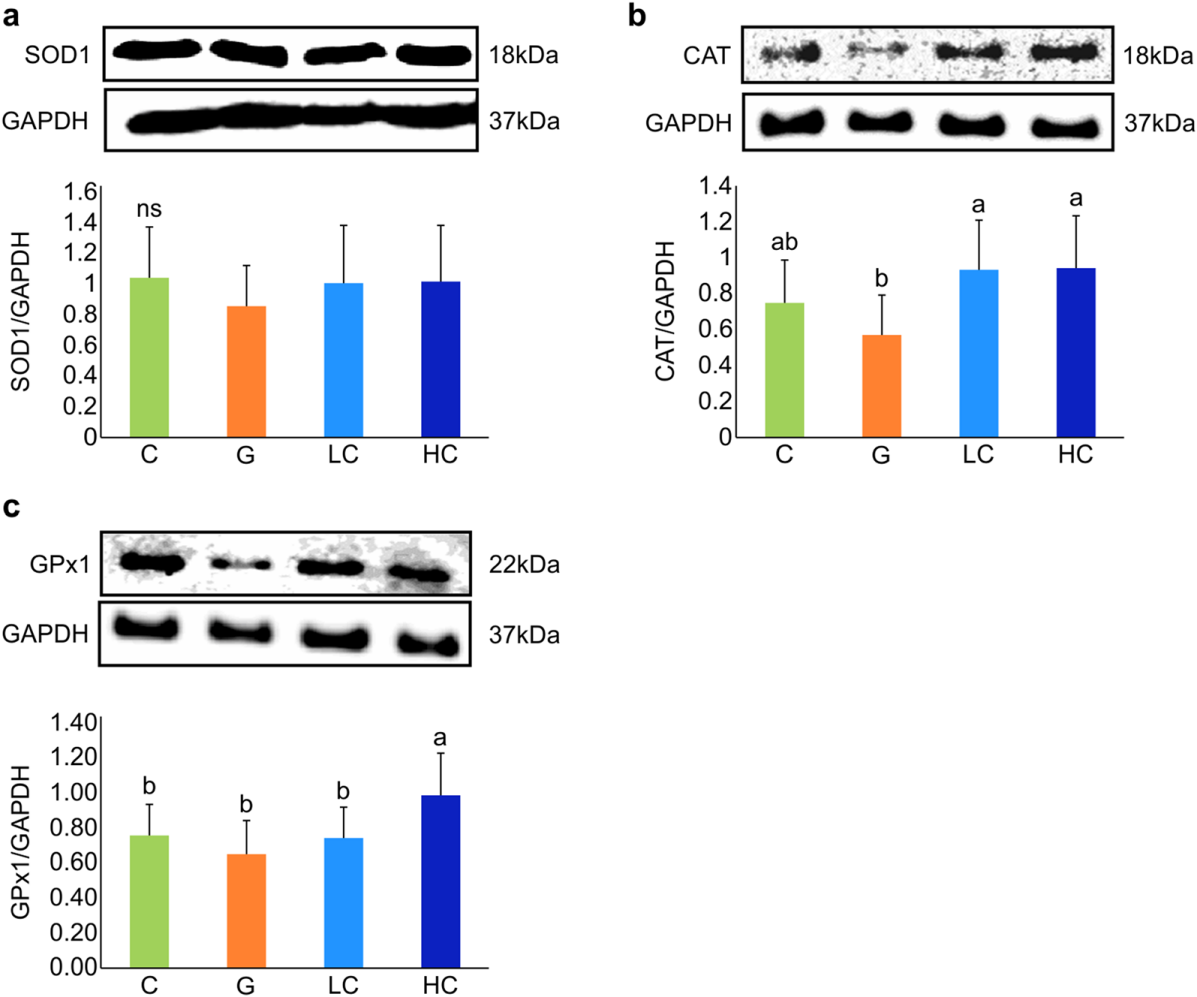
Effect of cacao on protein expression levels of antioxidant enzymes. (**a**) Expression levels of SOD1; (**b**) CAT; (**c**) GPx1. Values represent mean ± SD, n = 8. ns: not significant; Means for a variable with different letters (a, b) differ significantly (*p* < 0.05). C, control; G, d-galactose-induced aging; LC, d-galactose injection with 10% cacao powder mixed diet; HC, d-galactose injection with 16% cacao powder mixed diet.

Antioxidant enzymes such as SOD1, CAT, and GPx1 were investigated with western blot (Fig. 5). The expression level of SOD1 protein was lowest in the G group. Though the LC and HC groups showed increased expression levels, the increase was not significant. Moreover, the levels in the latter groups were relatively similar to the C group (Fig. 5a). Furthermore, the expression levels of CAT exhibited a significant increase in the LC and HC groups compared with the G group (*p* = 0.009); however, they were relatively similar to that in the C group (Fig. 5b). On the contrary, the expression level of GPx1 in the HC group was significantly higher as compared to the remaining three groups, whereas in the G group, it showed the lowest expression (*p* = 0.011) (Fig. 5c).

### Neuroprotective effect of cacao on PI3K/Akt mediated Caspase-3 pathway in the brain

**Figure 6:**
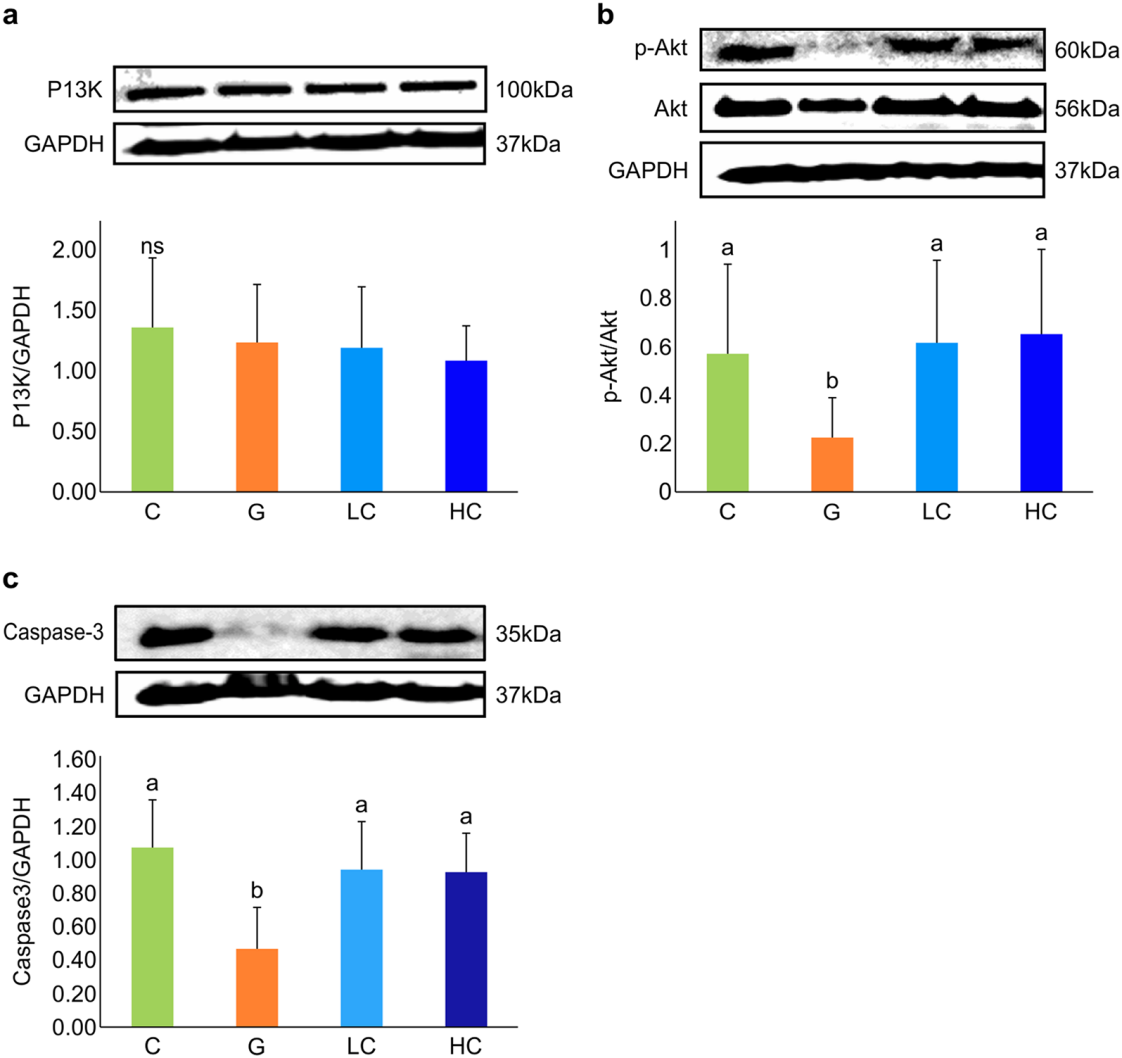
Effect of cacao supplementation on protein expression levels of neuroprotective enzymes. (**a**) Expression levels of PI3K; (**b**) the ratio of phospho-Akt/Akt levels; (**c**) expression levels of procaspase-3. Values represent mean ± SD, n = 8. ns: not significant; means for a variable with different letters (a, b) differ significantly (*p* < 0.05). C, control; G, d-galactose-induced aging; LC, d-galactose injection with 10% cacao powder mixed diet; HC, d-galactose injection with 16% cacao powder mixed diet.

PI3K and Akt act as anti-apoptotic signaling molecules mediating cell survival responses. The protein expression levels of these apoptosis-related enzymes are as shown in Fig. 6. PI3K did not show any significant difference between the studied groups (Fig. 6a). However, the ratio of phospho-Akt/Akt demonstrated a significant increase in the LC and HC groups compared with the G group (*p* = 0.044); LC, HC groups, and C group showed relatively the same levels, and a similar trend of the increase was also observed for procaspase-3 in the LC and HC groups compared to the G group, wherein the expression levels in LC and HC groups were similar to the C group (*p* = 0.001) (Fig. 6b, c).

## Discussion

Many theories have explained the process of aging over several years. The free radical theory has been supported by several studies with evidence that accumulation of oxidative damage accelerates aging and MDA depostion^24,25^. AGEs, the end product of glycation, binds with the AGE receptors, consequently increasing ROS^26^. Previous studies have shown that d-galactose can be used as a mimetic model of the aging process^13,14,27,28^. d-galactose is a form of reducing sugar, which produces AGEs when combined with free amines of amino acids^13^. It has been shown that chronic administration of d-galactose leads to ROS accumulation and increases the generation of free radicals^27,29^. The generated ROS is likely to cause mitochondrial dysfunction, oxidative stress, inflammation, and apoptosis in cells^28,30–32^. Cacao has long been studied as a beneficial natural antioxidant source with antioxidative, anti-inflammatory, anti-apoptotic, and anticarcinogenic properties^16,19–23,33,34^. The protective effects of cacao powder in the d-galactose-induced aging model were investigated for the first time in the present study. Here, we have shown that the cacao powder supplementation has protective effects against oxidative stress, cholinergic degeneration, and apoptosis in d-galactose induced aging rat model.

Previous studies have confirmed successful induction of aging in SD rats by injecting d-galactose at the age of 12 weeks and continuing the injection for 6 or 7 weeks^32,35^. Accordingly, d-galactose was first injected at the age of 12 weeks and continued for 12 weeks. The organ coefficients estimated for different organs, though, showed a tendency to decrease in the brain and kidney of the G group; the reduction was not significant. The liver and lung coefficients were almost the same in the C and G groups, but the coefficient of heart tended to increase in the G group though not significantly different. Earlier studies have reported that the organ-to-body weight ratio showed a tendency to decrease from week 13 to week 78 in most of the organs^36^. Moreover, it has also been explained that the increase in whole heart weight implies hypertrophy associated with high blood pressure or aging^37^. Therefore, in this study, though not all organ coefficients were consistent with previous studies, the observed tendencies indicated their possible role in the process of aging.

The results of the present study demonstrated that the levels of MDA in the serum and brain and the level of serum AGEs increased in group G, consistent with previous studies^15,27^. The increased levels of MDA and AGEs have been interpreted as the markers of aging^15,27,38^. Thus, the findings of the present study confirmed the successful induction of aging through the d-galactose-induced aging model. Cacao powder supplemented groups demonstrated attenuating effects on the levels of serum MDA, brain and liver MDA, and serum AGEs. These results indicate that chronic supplementation of polyphenol-rich cacao powder either delays or ameliorates the age-related changes.

Synaptic transmission can be improved by the stimulation of neurotransmitter availability, such as ACh in the synaptic cleft^39^. Ach is a critical neurotransmitter regulating cognitive functions like learning, memory and sleeping abilities^39^. AChE stimulates the degradation of ACh to acetate and choline^9^. Furthermore, previous researches also have shown increased AChE activity in d-galactose injected group, implying deterioration of cognitive decline in cholinergic neurons^9,40^. In the present study, the chronic administration of d-galactose showed a significant increase in AChE activity, thus suggesting impairment of the cholinergic system of the brain, which was observed to be ameliorated by the supplementation of cacao powder.

Antioxidant enzymes such as SOD, CAT, and GPx are the primary defense system preventing biological macromolecules from oxidative stress^41^. SODs convert superoxide anion to hydrogen peroxide, and then CAT and GPx degrade hydrogen peroxide to water^8^. SOD1, also known as SOD [Cu–Zn], is a free radical scavenging enzyme playing a major role in intracellular defense mechanisms. Some evidence support that SOD1 knockout models can shorten life span^41^. Moreover, reduced CAT activity have also been speculated to compromise the overall defense system of antioxidant enzymes. The results of the present study demonstrated a nonsignificant difference between the protein expression of SOD and GPx in the brain tissues of the C and G group rats. The high dose of cacao supplementation group only showed an increase in GPx protein expression. However, CAT showed a tendency of decrease in group G compared to group C, and cacao supplementation mitigated the reduction showing a significant increase of protein expression compared with G. These findings suggest a possible compensation reaction in the present experiment, compensating the decrease of one antioxidant by the increase of another antioxidant^14^. Moreover, a previous study also reported that age did not have significant impact on SOD and GPx activities in the brain^42^. Similarly, GPx activity has been speculated not to be associated with the age-related GSH level change, and the antioxidant enzymes may show an adaptive response to increased oxidative stress^14,42^. On the contrary, the CAT activity has been shown to decrease in the brain of the d-galactose treated group^13^. Collectively, though the reductions in the levels of studied antioxidant enzymes observed in this study were shown to be compensated by the cacao supplementation, further studies are required to validate its effects.

PI3K and Akt enzymes play a key role as major intracellular signaling enzymes involved in cell growth, proliferation, metabolism, and survival^4^. Akt is a necessary enzyme involving in the regulation of apoptosis suppression being phosphorylated and activated. Zhao et al.^38^ reported significant downregulation of the phosphorylation of PI3K and Akt in the brain by d-galactose treatment. Consistent with this study, the present study also demonstrated that the phosphorylation of Akt in the brain tissue of the G group was reduced, while cacao supplementation alleviated the reduction. This data indicates the potential of cacao in ameliorating the tissue damage incurred by apoptosis. However, PI3K did not show a significant difference between the groups, indicating a PI3K-independent mechanism for Akt activation, such as Integrin-linked kinase (ILK)^43^.

Caspases are activated via JNK (c-Jun-N-terminal kinase), and the extrinsic ‘death receptor’ pathway merges with the intrinsic pathway resulting in apoptosis. Of the caspase family, caspase-3, the downstream target of the PI3K/Akt pathway, is considered to be the most effective apoptotic protein^44^. Procaspase-3 was analyzed by western blot analysis of brain tissues to confirm whether this marker involves in the protective mechanism of cacao against d-galactose-induced apoptosis. Our data showed a significant decrease in procaspase-3 expression in the G group, and cacao supplementation countered this reduction. Similar to the present finding, an earlier study has shown a significant decrease in procaspase-3 protein expression in d-galactose treated group^44^, whereas contradictory reports are also available that demonstrated a significant increase in its expression in the d-galactose-induced group^45^. Taken together, the findings of the present study suggest that reduced procaspase-3 may have caused an increase of cleaved caspase-3, activating the cleavage of the cells.

The results were not dose-dependent between the two doses of cacao supplementation in each experiment. Serum MDA, AGE, brain and liver MDA all showed decreased levels in LC, HC compared to G. Serum MDA level decreased the most in LC while LC and HC did not show significantly different results in serum AGE, liver and brain MDA levels. AChE activity decreased the most in the LC group among the 4 groups. The protein expression of antioxidant enzyme CAT showed an insignificant difference between LC and HC. However, GPx1 demonstrated significantly increased protein expression in HC compared with LC. The protein expression of p-Akt/Akt and procaspase-3 all displayed similar results in LC and HC.

Taken together, the present study provides evidence that cacao supplementation enhances the protecting ability against oxidative stress, cholinergic impairment, and apoptosis induced by d-galactose in the rat brain. Our study confirmed d-galactose-induced aging is associated with brain MDA, MDA and AGE of serum. We also showed that cacao treatment improved CAT, GPx1, p-Akt/Akt, and procaspase-3 protein expression. Thus, cacao can be considered as an effective modulator of aging.

The present study was designed with only two different doses of cacao; therefore, there is a limitation determining the most effective dose by our study. Further studies are warranted to try out multiple doses of cacao powder supplementation to produce diverse results enabling the proposal of a more specific conclusion. Nevertheless, our observation presents a new mechanism of protecting the aging brain by chronic supplementation of cacao powder which can be of help in developing a more improved neuroprotecive agent.

## Conclusions

**Figure 7:**
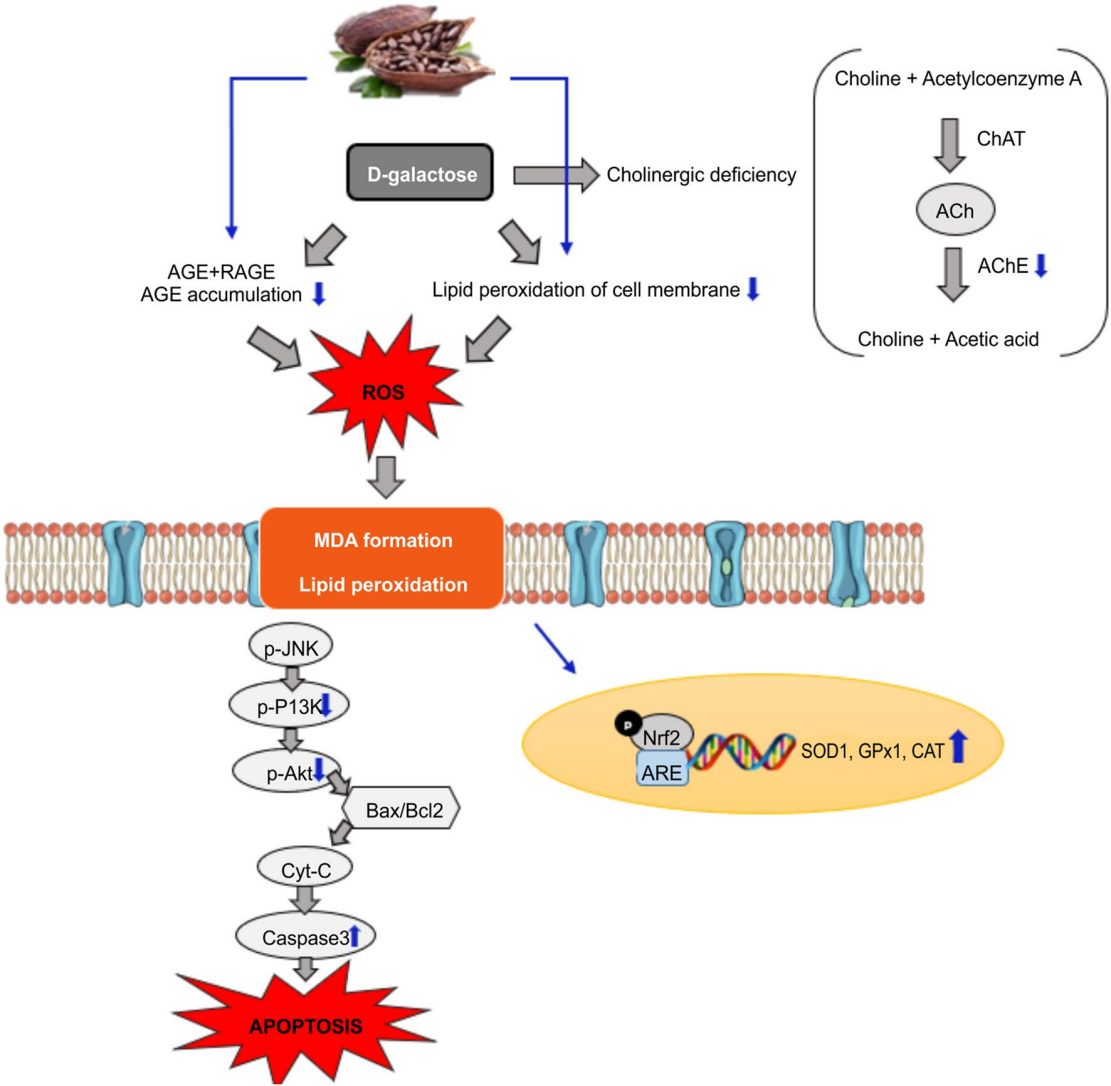
Hypothetical mechanism of d-galactose-induced aging model and the effects of cacao treatment in the brain and liver of SD rats.

Overall, the present study showed that d-galactose induced oxidative damage, cholinergic impairment, and apoptosis. Our study was designed based on the hypothesis of cacao’s protective mechanism (Fig. 7). Consumption of cacao showed improvements in the impairments induced by d-galactose in SD rats with activation of Akt mediated caspase-3 pathway and CAT in the brain and inactivation of AChE. Therefore, our study suggests that cacao powder may be an effective candidate as a therapuetic target for healty aging of the brain.

## Materials and methods

### Preparation of diets

Raw cacao powder was purchased from Navitas organics (California, United States) and sourced from Peru. We prepared the less processed raw cacao powder to avoid the loss of polyphenols during roasting or processing^46^. Its composition was investigated by SGS Korea Co., Ltd. before using it for experimental diets (Supplementary Table S1). The composition of cacao powder supplemented diets was modified from the AIN-93 M diet (Research Diet, New Brunswick, NJ, USA) and expressed with the percentage of cacao’s weight per total weight of the diet. The percentages of cacao powder were determined based on the previous studies^16,21,47,48^. The control group (C) and the d-galactose-induced aging group (G) were provided with the normal diet (with the same nutritional composition as AIN-93 M; Todo Bio), whereas LC and HC diets were made with 10% and 16% of cacao powder each. The amount of macronutrients and crude fiber contents were adjusted to be equivalent in all diets. The composition of each experimental diet is presented in Supplementary Table S2.

### Animals and treatments

Thirty-two 9-week-old male Sprague–Dawley (SD) rats, weighing 378–386 g, were obtained from Daehanbio, Gyeonggi-do, Korea. The animals were housed in a standard metal cage (3 per cage) under controlled temperature (21 ± 1 °C), humidity (50–60%) and 12 h dark/light cycle photoperiod throughout the study. They had access to water and the diet ad libitum. All experimental procedures were approved by the Animal Ethical Committee of Sookmyung Women’s University for the care and use of laboratory animals (SMWU-IACUC-1806-014). The study was carried out in compliance with all relevant guidelines including the ARRIVE guidelines. The animals were given 3 weeks of acclimation period to conduct aging induction on 12-week-old rats. The rats were randomly divided with a completely randomized design into four groups as follows (8 rats in each group):

- C—Control group fed with normal diet
- G—d-galactose injection with normal diet
- LC—d-galactose injection with a modified diet (supplemented with 10% cacao powder)
- HC—d-galactose injection with a modified diet (supplemented with 16% cacao powder)

All groups except those in the C group received d-galactose injection every day for 12 weeks. Group C was treated with 3 mL of saline (0.9% NaCl) injection per kg body weight intraperitoneally, whereas the remaining 3 groups were intraperitoneally injected with 3 mL of d-galactose (300 mg dissolved in 3 mL of saline) per kg bodyweight. All groups received the same normal diet for the first 4 weeks, and cacao powder mixed diets were provided to LC, HC groups for the last 8 weeks. Food intake was recorded every alternative day, and the body weight was recorded once a week with an electronic balance (OHAUS, NJ, USA).

### Serum and tissue preparation

The animals were fasted for 24 hours, final body weights were weighed, and all animals were euthanized with CO_2_. Brains, livers, kidneys, hearts, and lungs were immediately isolated and weighed. The blood samples collected by cardiopuncture were centrifuged at 1912 RCF for 30 min (Combi-514R, Hanil Co.Ltd., Seoul, Korea) to collect the serum for biochemical analyses. The brain and liver tissues were homogenized in phosphate-buffered saline (PBS) (P5244, Sigma-Aldrich, Co., USA) or PRO-PREP kit (17081, iNtRON Biotechnology, Gyeonggi-do, Korea) and centrifuged at 1912 RCF, 15 min × 3 times, at 4 °C). Removed brains were frozen in liquid nitrogen and were stored at − 70 °C (DF8517; Ilshin Laboratory Co., Ltd., Seoul, Korea) until analysis. All serum and tissues other than brains were kept on ice and stored at − 70 °C for further analysis.

### Calculation of organ coefficients

The weights of the following organs were measured with electronic balance (OHAUS, NJ, USA) after sacrificing the animals and isolating the organs—brains, livers, kidneys, hearts, and lungs. The final body weights of the rats were measured on the day before sacrifice, and coefficients of each organ were calculated using the following equation^10^;

$${\text{Coefficient }}\,{\text{of}}\,{\text{organ }}\left( {{\text{g}}/100{\text{ g}}} \right) = \frac{{{\text{Organ}}\,{\text{weight}}\,\left( {\text{g}} \right)}}{{{\text{Body}}\,{\text{weight}}\,\left( {\text{g}} \right){ }}} \times 100$$

### Oxidative stress measurements

Serum MDA, AGEs, liver and brain MDA levels were measured to assess the oxidative stress status of each group. The levels of serum MDA (μM) and AGEs (μg/mL) were measured using the MDA ELISA Kit (E-EL-0060, Elabscience, USA) and the AGEs ELISA Kit (E-EL-M0359, Elabscience, USA), respectively following the instructions of manufacturers. Thiobarbituric acid (TBA) (BDH Chemicals Ltd., England) was used in measuring brain and liver MDA levels, as described in a previous study^49^. Briefly, 0.1 g of brain and liver samples, each was homogenized with 1 mL of PBS. Then 0.2 mL of 8.1% sodium dodecyl sulfate solution (SDS) (IBS-BS003a, iNtRON Biotechnology, Gyeonggi-do, Korea), 1.5 mL of 20% acetic acid (DUKSAN, Gyeonggi-do, Korea), and 1.5 mL of 0.8% aqueous TBA were added to the sample homogenates in order. After proper mixing, the samples were heated in the water bath (Chang Shin Science Co., Gyeonggi-do, Korea) at 95 °C for 1 h. The heated mixtures were cooled with tap water and ice. Subsequently, 1 mL distilled water and 5 mL n-butanol (SAMCHUN, Gyeonggi- do, Korea) were added to the samples successively and vortexed. They were then centrifuged for 20 min at 3399 RCF (Combi-514R) to obtain a clear, organic layer. Then, the clear layer was transferred to 96-well plates, and the absorbance was measured with an ELISA plate reader at 532 nm (Epoch Microplate Spectrophotometer, Biotek Inc., Winooski, Vermont, USA). The MDA contents of brain and liver samples thus obtained were presented in nmol MDA per g of tissues. The standard curve was developed using 1,1,3,3-tetraethoxypropane (108,383, Sigma-Aldrich, Co., USA).

### Acetylcholinesterase (AChE) activity measurement

Cholinergic system impairment of brain tissue was detected using the Acetylcholinesterase (AchE) Activity Assay Kit (E-BC-K174, Elabscience, USA), which is based on the principle that AchE catalyzes the hydrolysis of acetylcholine to form choline, then choline reacts with dithio p-nitrobenzoic acid (DTNB) and forms 5-mercapto-nitrobenzoid acid (TNB). TNB has an absorption peak at 412 nm. Here, we analyzed the activity of AchE by assessing the increasing rate of absorbance at 412 nm. All experiment procedures were carried out following the manufacturer’s instructions. The protein concentrations of the samples (mg/mL) were analyzed with the PRO-MEASURE™ kit (21011, iNtRON Biotechnology, Gyeonggi-do, Korea). The detected increasing rate of absorbance at 412 nm was calculated with the following formula;

$${\text{AchE}}\,{\text{activity }}\left( {{\text{nmol/min/mg }}\,{\text{prot}}} \right) = \left( {\Delta {\text{A}} \times \frac{{V_{Total} }}{{{\upvarepsilon } \times {\text{d}}}} \times 10^{9} } \right) \div \left( {Cpr \times V_{sample} } \right) \div T = 490 \times \Delta {\text{A}} \div {\text{Cpr}}$$

where, ΔA = A2-A1, the difference between the OD values at 10 s (A1) and 190 s (A2); V_Total_: total volume of the reaction system, 2 × 10^–4^ L; ε: molar extinction coefficient of TNB, 13.6 × 10^3^ L/mol/cm; d: the optical path of the 96-well microplate, 0.5 cm; V_sample_: volume of sample added into the reaction system, 20 μL = 2 × 10^–2^ mL; T: The reaction time, 3 min.; Cpr: the concentration of protein in the sample, mg/mL.

### Western blot analysis

To evaluate expression levels of the antioxidant and apoptosis-related proteins in brain tissues, 0.01–0.03 g of brain tissue was homogenized with 0.5–0.8 mL of Pro-prep protein extraction solution (17081, iNtRON Biotechnology, Gyeonggi-do, Korea) on ice. The samples were centrifuged at 1912 RCF (15 min × 3 times, 4 °C), and the supernatant was collected for further analysis. The concentrations of the isolated proteins were estimated using the PRO-MEASURE™ kit (21011, iNtRON Biotechnology, Gyeonggi-do, Korea). Brain samples (20–75 μg) and GangNam-STAIN™ Prestained Protein Ladder (24052, iNtRON Biotechnology, Gyeonggi-do, Korea), a protein marker, were loaded on an SDS-PAGE gel. The separated proteins were then electrophoretically (Bio-Rad Laboratories, Inc., Hercules, CA, USA) transferred onto the PVDF membrane (Merck Millipore, MA, USA), activated in methanol for 1 min. Subsequently, the membrane was blocked for 1 h using a blocking buffer containing 5% skim milk, 1X PBS and 0.1% Tween-20 (PBS-T). The membrane was then incubated with the diluted primary antibodies at 4 °C for 3 h to 15 h, depending on the antibody. The primary antibodies were SOD1 polyclonal antibody (1:1000, Invitrogen, California, USA), CAT polyclonal antibody (1:500, Cell Signaling, Technology, Inc., Massachusetts, USA), Gpx1 polyclonal antibody (1:1000, Abcam, Cambridge, UK), PI3 Kinase Class III antibody (1:800, Cell Signaling Technology, Inc., Massachusetts, USA), Akt polyclonal antibody (1:800, Abnova, Taipei, Taiwan), phospho-Akt (Ser473) antibody (1:200, Cell Signaling Technology, Inc., Massachusetts, USA), and Caspase-3 antibody (1:500, Cell Signaling Technology, Inc., Massachusetts, USA). After incubation, the membrane was washed 3 times (10 min each) with TBS-T followed by incubation with Mouse Anti-Rabbit IgG H&L (HRP) secondary antibody (1:5000, Santa Cruz Biotechnology Inc., Texas, USA) at 4 °C for 1–2 h. The membranes were then washed thrice (@10 min) with TBS-T. The protein expression was normalized to the expression of the control protein, GAPDH (1:1000, Cell Signaling Technology, Inc., Massachusetts, USA). Miracle-Star™ Western Blot Detection System (16028, iNtRON Biotechnology, Gyeonggi-do, Korea) was used to detect chemiluminescence. The immunoreactive band intensities were visualized by densitometric analysis (LAS-3000, Fujifilm Co., Tokyo, Japan).

### Statistical analysis

Statistical analysis was performed with IBM SPSS Statistics 23 (SPSS Inc., Chicago, IL, USA). All data are presented as means ± standard deviations (SD). One-way ANOVA was used to compare the mean values between groups, and Duncan’s multiple tests were performed for multiple comparisons. A *p* value of < 0.05 was considered statistically significant.

## Supplementary Information

Supplementary Information 1.

Supplementary Information 2.

## Data Availability

All data generated or analyzed during this study are included in this published article (and its Supplementary Information files).

## References

[1] Liang J (2019). *Dendrobium officinale* polysaccharides attenuate learning and memory disabilities via anti-oxidant and anti-inflammatory actions. Int. J. Biol. Macromol..

[2] Kawakami K, Kadota J, Iida K, Shirai R, Abe K, Kohno S (1999). Reduced immune function and malnutrition in the elderly. Tohoku. J. Exp. Med..

[3] Beckman KB, Ames BN (1998). The free radical theory of aging matures. Physiol. Rev..

[4] Shah SA, Lee HY, Bressan RA, Yun DJ, Kim MO (2014). Novel osmotin attenuates glutamate-induced synaptic dysfunction and neurodegeneration via the JNK/PI3K/Akt pathway in postnatal rat brain. Cell Death Dis..

[5] Kitagishi Y, Nakanishi A, Ogura Y, Matsuda S (2014). Dietary regulation of PI3K/AKT/GSK-3beta pathway in Alzheimer’s disease. Alzheimers Res. Ther..

[6] Choi YJ (2012). Attenuation of age-related changes in FOXO3a activity and the PI3K/Akt pathway by short-term feeding of ferulate. Age (Dordr).

[7] He M, Zhao L, Wei MJ, Yao WF, Zhao HS, Chen FJ (2009). Neuroprotective effects of (-)-epigallocatechin-3-gallate on aging mice induced by d-galactose. Biol. Pharm. Bull..

[8] Finkel T, Holbrook NJ (2000). Oxidants, oxidative stress and the biology of ageing. Nature.

[9] Qu Z (2016). Protective effect of tetrahydropalmatine against d-galactose induced memory impairment in rat. Physiol. Behav..

[10] Jeong H, Liu Y, Kim HS (2017). Dried plum and chokeberry ameliorate d-galactose-induced aging in mice by regulation of Pl3k/Akt-mediated Nrf2 and Nf-kB pathways. Exp. Gerontol..

[11] Ames BN, Shigenaga MK, Hagen TM (1993). Oxidants, antioxidants, and the degenerative diseases of aging. Proc. Natl. Acad. Sci. U.S.A..

[12] Shwe T, Pratchayasakul W, Chattipakorn N, Chattipakorn SC (2018). Role of d-galactose-induced brain aging and its potential used for therapeutic interventions. Exp. Gerontol..

[13] Chen P, Chen FC, Zhou BH (2018). Antioxidative, anti-inflammatory and anti-apoptotic effects of ellagic acid in liver and brain of rats treated by d-galactose. Sci. Rep..

[14] Hadzi-Petrushev N, Stojkovski V, Mitrov D, Mladenov M (2015). d-galactose induced changes in enzymatic antioxidant status in rats of different ages. Physiol. Res..

[15] Li YN (2014). Saponins from *Aralia taibaiensis* attenuate d-galactose-induced aging in rats by activating FOXO3a and Nrf2 pathways. Oxid. Med. Cell. Longev..

[16] Fernandez-Millan E (2015). Cocoa-rich diet attenuates beta cell mass loss and function in young Zucker diabetic fatty rats by preventing oxidative stress and beta cell apoptosis. Mol. Nutr. Food. Res..

[17] Patel AK, Rogers JT, Huang XD (2008). Flavanols, mild cognitive impairment, and alzheimer’s dementia. Int. J. Clin. Exp. Med..

[18] Vauzour D, Vafeiadou K, Rodriguez-Mateos A, Rendeiro C, Spencer JPE (2008). The neuroprotective potential of flavonoids: A multiplicity of effects. Genes Nutr..

[19] Spencer JPE (2009). Flavonoids and brain health: Multiple effects underpinned by common mechanisms. Genes Nutr..

[20] Lee KW, Kim YJ, Lee HJ, Lee CY (2003). Cocoa has more phenolic phytochemicals and a higher antioxidant capacity than teas and red wine. J. Agric. Food Chem..

[21] Cady RJ, Durham PL (2010). Cocoa-enriched diets enhance expression of phosphatases and decrease expression of inflammatory molecules in trigeminal ganglion neurons. Brain Res..

[22] Shah ZA (2010). The flavanol (-)-epicatechin prevents stroke damage through the Nrf2/HO1 pathway. J. Cereb. Blood Flow Metab..

[23] Fernandez-Fernandez L (2012). LMN diet, rich in polyphenols and polyunsaturated fatty acids, improves mouse cognitive decline associated with aging and Alzheimer’s disease. Behav. Brain. Res..

[24] Muller FL, Lustgarten MS, Jang Y, Richardson A, Van Remmen H (2007). Trends in oxidative aging theories. Free Radic. Biol. Med..

[25] Dei R (2002). Lipid peroxidation and advanced glycation end products in the brain in normal aging and in Alzheimer's disease. Acta Neuropathol..

[26] Perrone A, Giovino A, Benny J, Martinelli F (2020). Advanced glycation end products (AGEs): Biochemistry, signaling, analytical methods, and epigenetic effects. Oxid. Med. Cell. Longev..

[27] Doan VM (2015). Yulangsan polysaccharide improves redox homeostasis and immune impairment in d-galactose-induced mimetic aging. Food Funct..

[28] Banji OJ, Banji D, Ch K (2014). Curcumin and hesperidin improve cognition by suppressing mitochondrial dysfunction and apoptosis induced by d-galactose in rat brain. Food Chem. Toxicol..

[29] Turgut NH (2016). Effect of black mulberry (*Morus nigra*) extract treatment on cognitive impairment and oxidative stress status of d-galactose-induced aging mice. Pharm. Biol..

[30] Kumar A, Dogra S, Prakash A (2009). Effect of carvedilol on behavioral, mitochondrial dysfunction, and oxidative damage against d-galactose induced senescence in mice. Naunyn-Schmiedeberg’s Arch. Pharmacol..

[31] Prakash A, Kumar A (2013). Pioglitazone alleviates the mitochondrial apoptotic pathway and mito-oxidative damage in the d-galactose-induced mouse model. Clin. Exp. Pharmacol. Physiol..

[32] Rehman SU, Shah SA, Ali T, Chung JI, Kim MO (2017). Anthocyanins reversed d-galactose-induced oxidative stress and neuroinflammation mediated cognitive impairment in adult rats. Mol. Neurobiol..

[33] Rusconi M, Conti A (2010). Theobroma cacao L. the Food of the Gods: A scientific approach beyond myths and claims. Pharmacol. Res..

[34] Rozan P, Hidalgo S, Nejdi A, Bisson JF, Lalonde R, Messaoudi M (2007). Preventive antioxidant effects of cocoa polyphenolic extract on, free radical production and cognitive performances after heat exposure in Wistar rats. J. Food Sci..

[35] Zhu J (2014). Ginsenoside Rg1 prevents cognitive impairment and hippocampus senescence in a rat model of d-galactose-induced aging. PLoS ONE.

[36] Piao Y, Liu Y, Xie X (2013). Change trends of organ weight background data in sprague dawley rats at different ages. J. Toxicol. Pathol..

[37] Azman KF, Zakaria R (2019). d-Galactose-induced accelerated aging model: an overview. Biogerontology.

[38] Zhao MH, Tang XQ, Gong DY, Xia P, Wang FS, Xu SJ (2020). Bungeanum improves cognitive dysfunction and neurological deficits in d-galactose-induced aging mice via activating PI3K/Akt/Nrf2 signaling pathway. Front. Pharmacol..

[39] Perez-Lloret S, Barrantes FJ (2016). Deficits in cholinergic neurotransmission and their clinical correlates in Parkinson's disease. NPJ Parkinsons Dis..

[40] Mohapel P, Leanza G, Kokaia M, Lindvall O (2005). Forebrain acetylcholine regulates adult hippocampal neurogenesis and learning. Neurobiol. Aging.

[41] Deepa SS (2017). A new mouse model of frailty: The Cu/Zn superoxide dismutase knockout mouse. GeroScience.

[42] Tiana L, Caib Q, Wei H (1998). Alterations of Antioxidant Enzymes and Oxidative Damage to Macromolecules in Different Organs of Rats During Aging. Free Radic. Biol. Med..

[43] Gary DS, Milhavet O, Camandola S, Mattson MP (2003). Essential role for integrin linked kinase in Akt-mediated integrin survival signaling in hippocampal neurons. J. Neurochem..

[44] Ou Y, Yuan Z, Li K, Yang X (2012). Phycocyanin may suppress d-galactose-induced human lens epithelial cell apoptosis through mitochondrial and unfolded protein response pathways. Toxicol. Lett..

[45] Hsieh HM, Wu WM, Hu ML (2009). Soy isoflavones attenuate oxidative stress and improve parameters related to aging and Alzheimer's disease in C57BL/6J mice treated with d-galactose. Food Chem. Toxicol..

[46] Oracz J, Zyzelewicz D, Nebesny E (2015). The content of polyphenolic compounds in cocoa beans (*Theobroma cacao* L.), depending on variety, growing region, and processing operations: A review. Crit. Rev. Food Sci. Nutr..

[47] Granado-Serrano AB, Martin MA, Bravo L, Goya L, Ramos S (2009). A diet rich in cocoa attenuates N-nitrosodiethylamine-induced liver injury in rats. Food Chem. Toxicol..

[48] Yamade T, Yamada Y, Okano Y, Terashima T, Yokogoshi H (2009). Anxiolytic effects of short- and long-term administration of cacao mass on rat elevated T-maze test. J. Nutr. Biochem..

[49] Ohkawa H, Ohishi N, Yagi K (1979). Assay for lipid peroxides in animal tissues by thiobarbituric acid reaction. Anal. Biochem..

[50] Deng S (2019). Molecular basis of neurophysiological and antioxidant roles of Szechuan pepper. Biomed. Pharmacother..

[51] Magrone T, Russo MA, Jirillo E (2017). Cocoa and dark chocolate polyphenols: From biology to clinical applications. Front. Immunol..

