# Supplementary material for: Cacao powder supplementation attenuates oxidative stress, cholinergic impairment, and apoptosis in d-galactose-induced aging rat brain
Source: Sci Rep. 2021 Sep 9;11:17914. doi: 10.1038/s41598-021-96800-y (PMC8429651; doi:10.1038/s41598-021-96800-y)
Supplement: Supplementary file 1 — Supplementary Information 1. [file 41598_2021_96800_MOESM1_ESM.pdf]

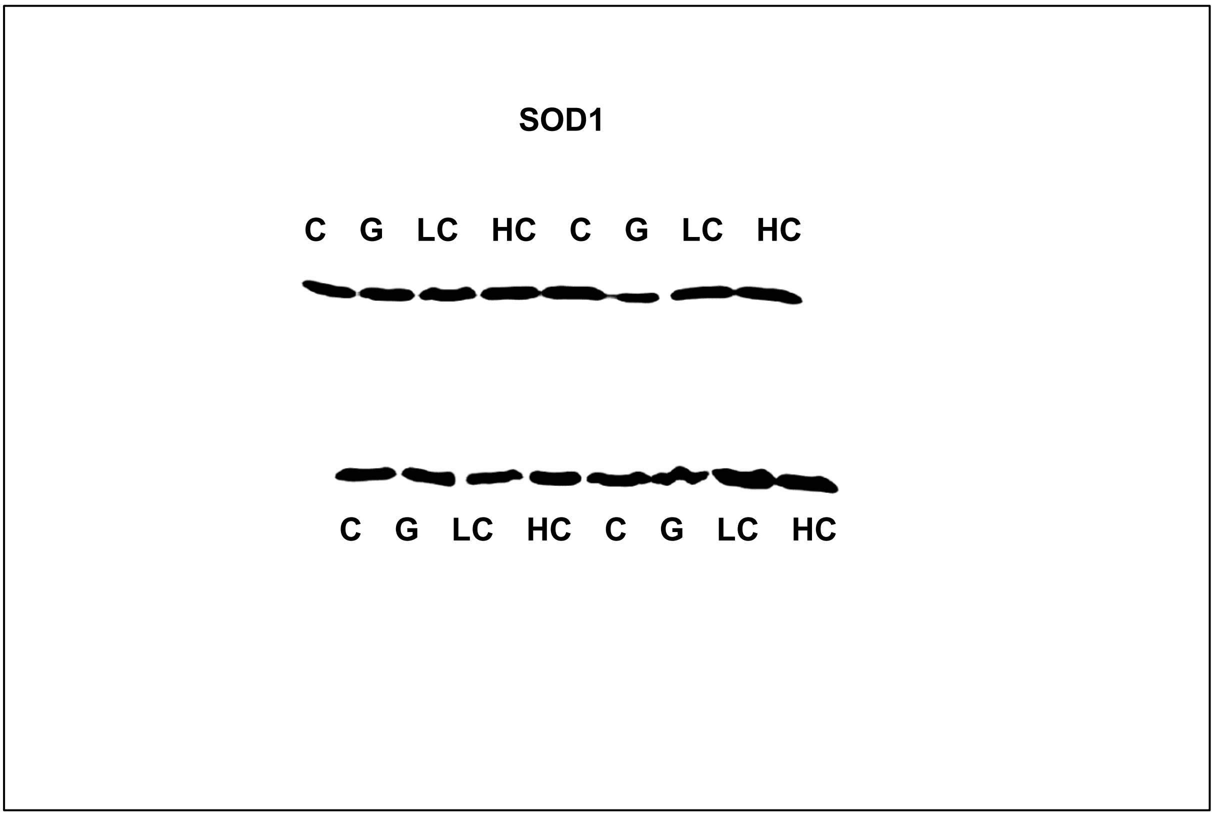
SOD1
C G LC HC C G LC HC
C G LC HC C G LC HC

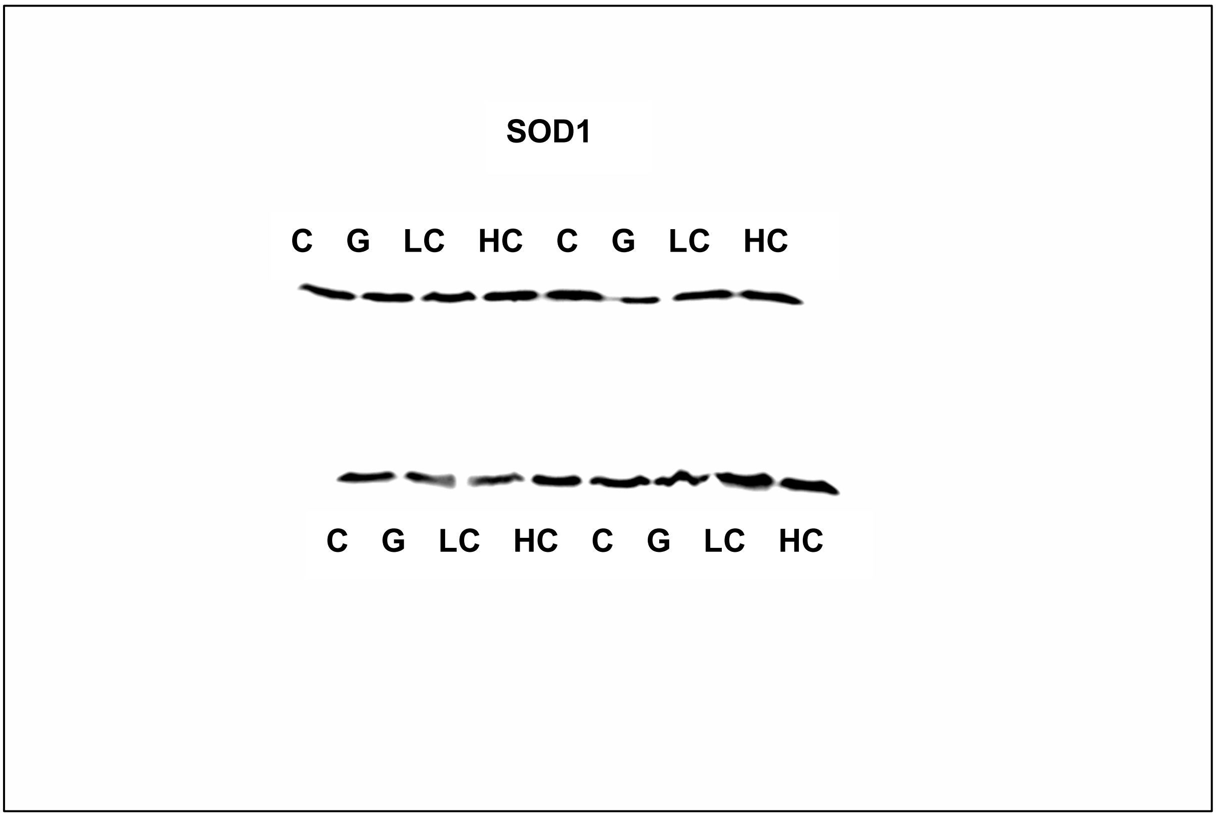
SOD1
C G LC HC C G LC HC
C G LC HC C G LC HC

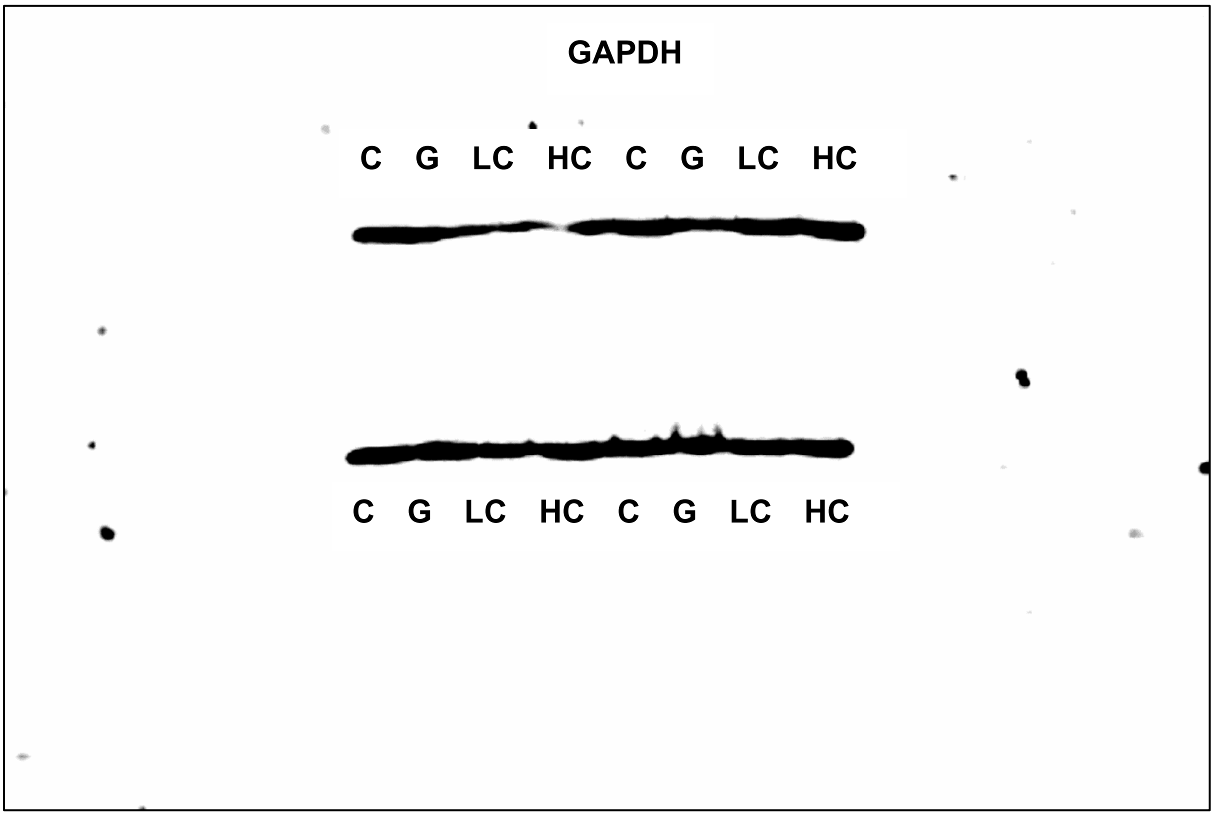
GAPDH
C G LC HC C G LC HC
C G LC HC C G LC HC

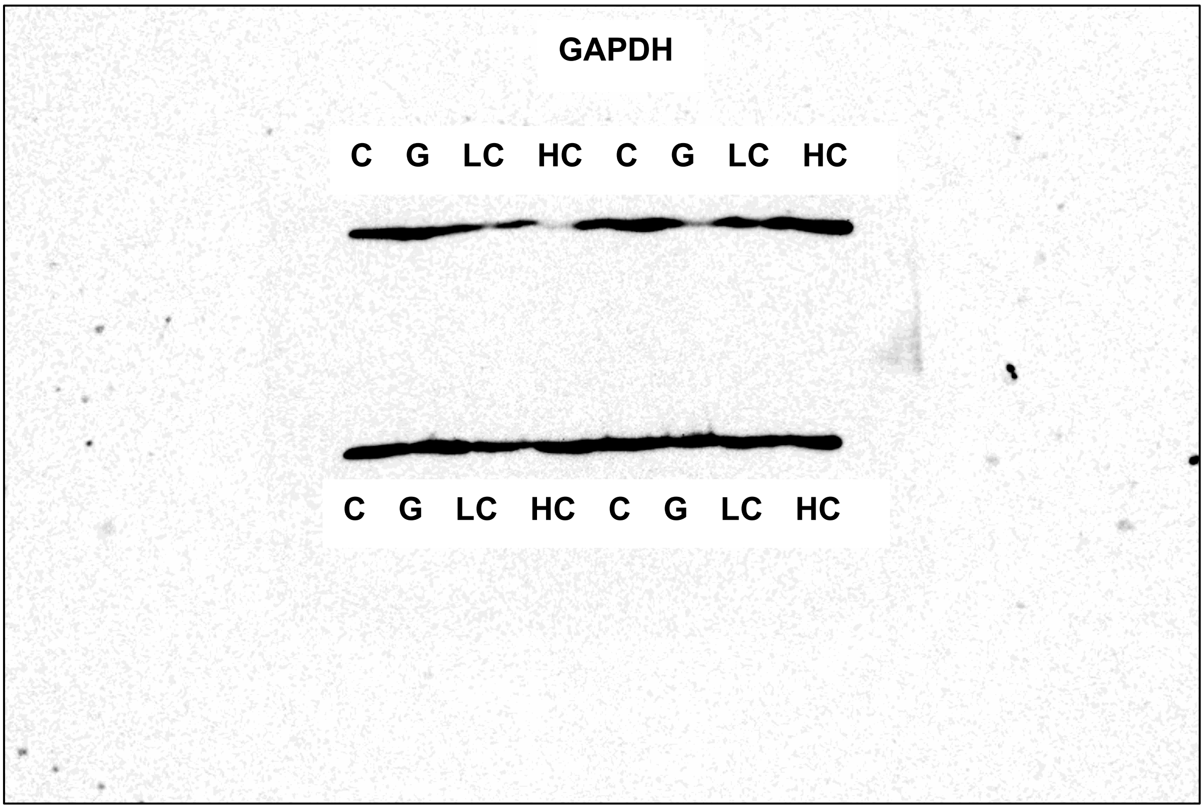
GAPDH
C G LC HC C G LC HC
C G LC HC C G LC HC

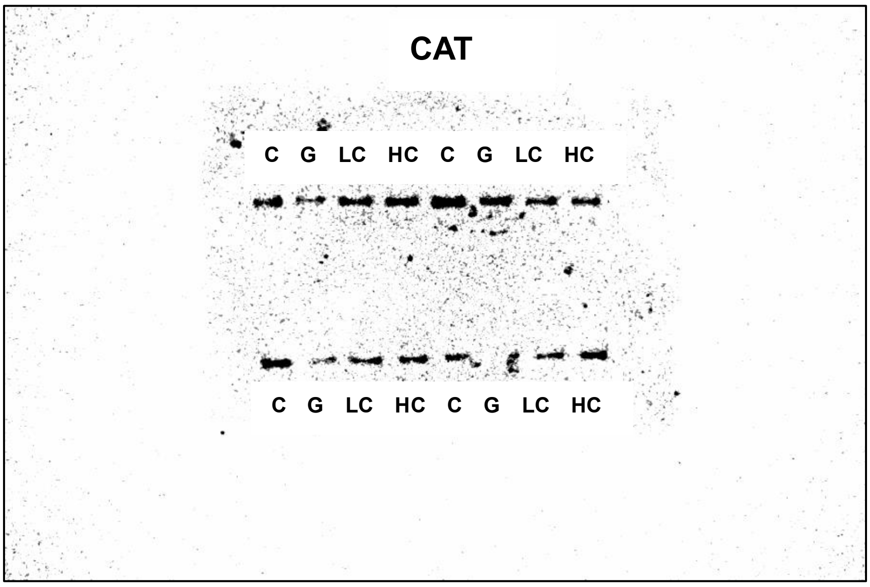
CAT
C G LC HC C G LC HC
C G LC HC C G LC HC

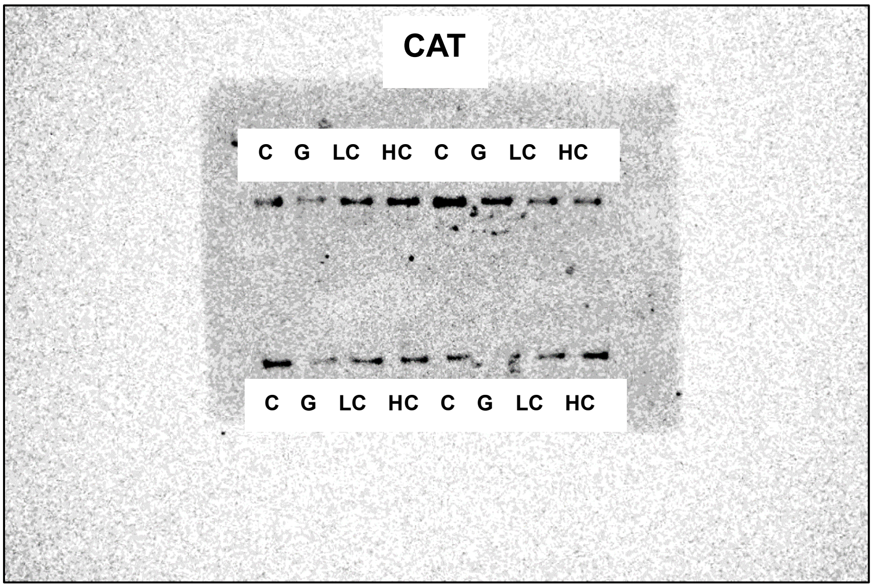
CAT
C G LC HC C G LC HC
C G LC HC C G LC HC

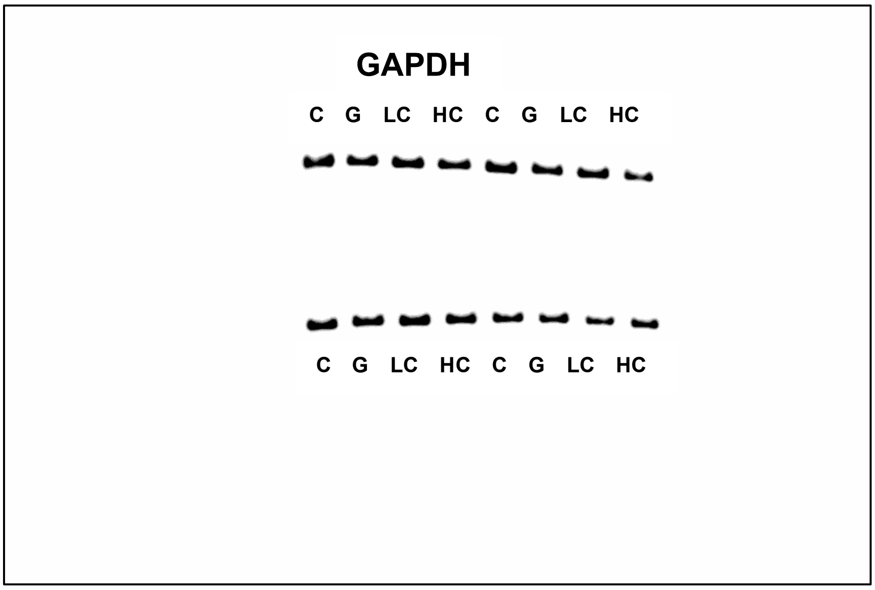
GAPDH
C G LC HC C G LC HC
C G LC HC C G LC HC

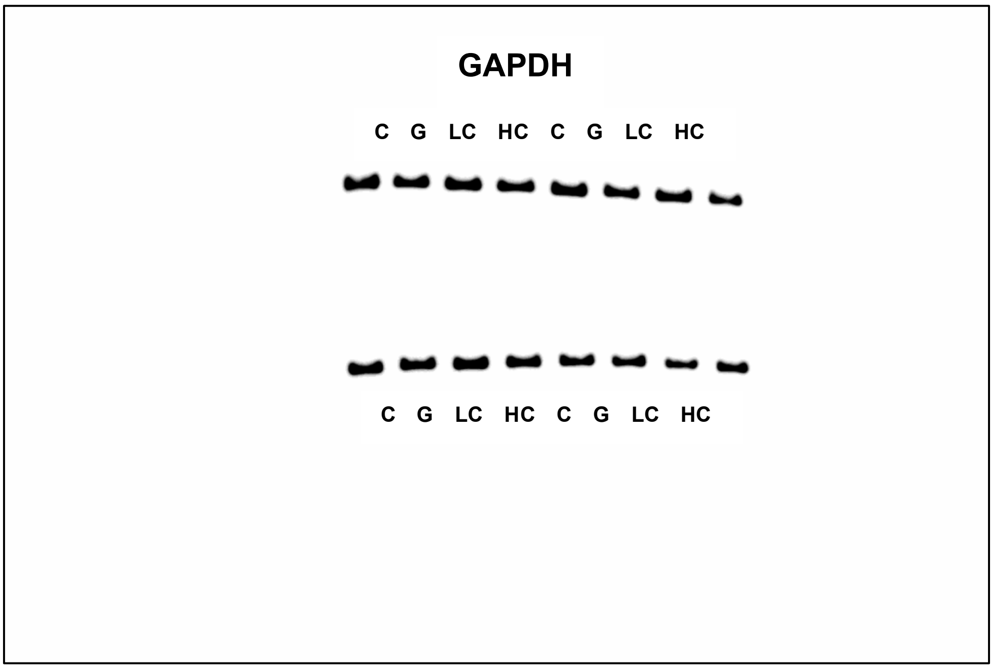
GAPDH
C G LC HC C G LC HC
C G LC HC C G LC HC

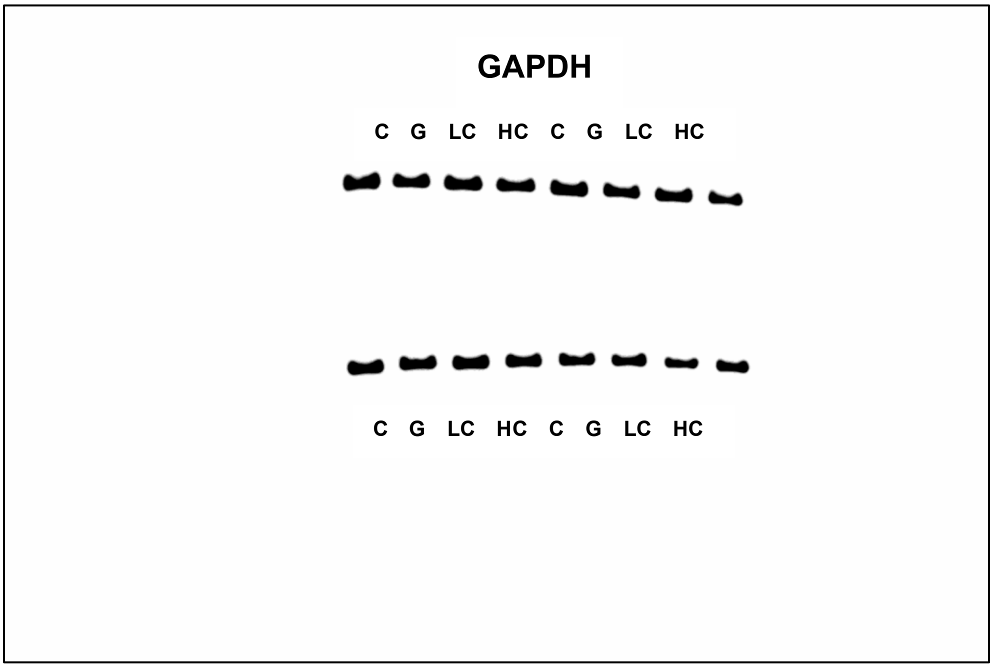
GAPDH
C G LC HC C G LC HC
C G LC HC C G LC HC

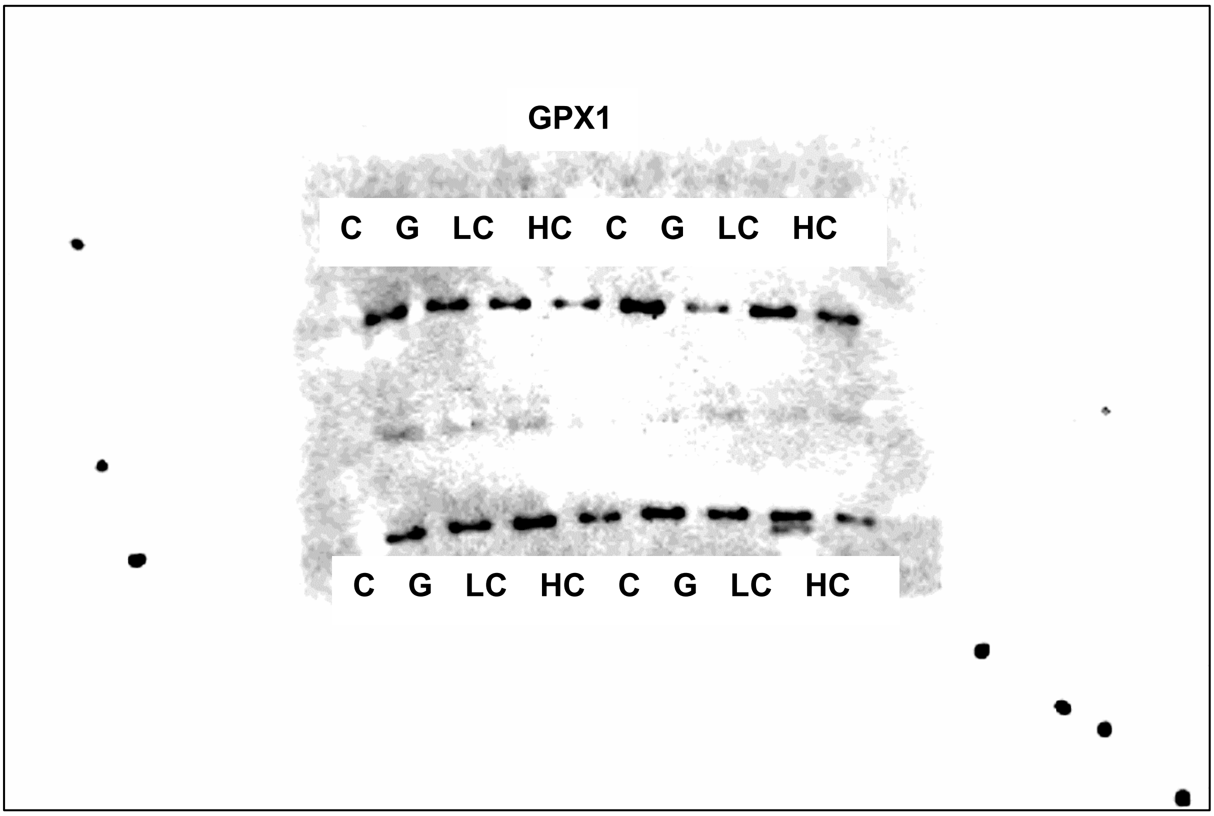
GPX1
C G LC HC C G LC HC
C G LC HC C G LC HC

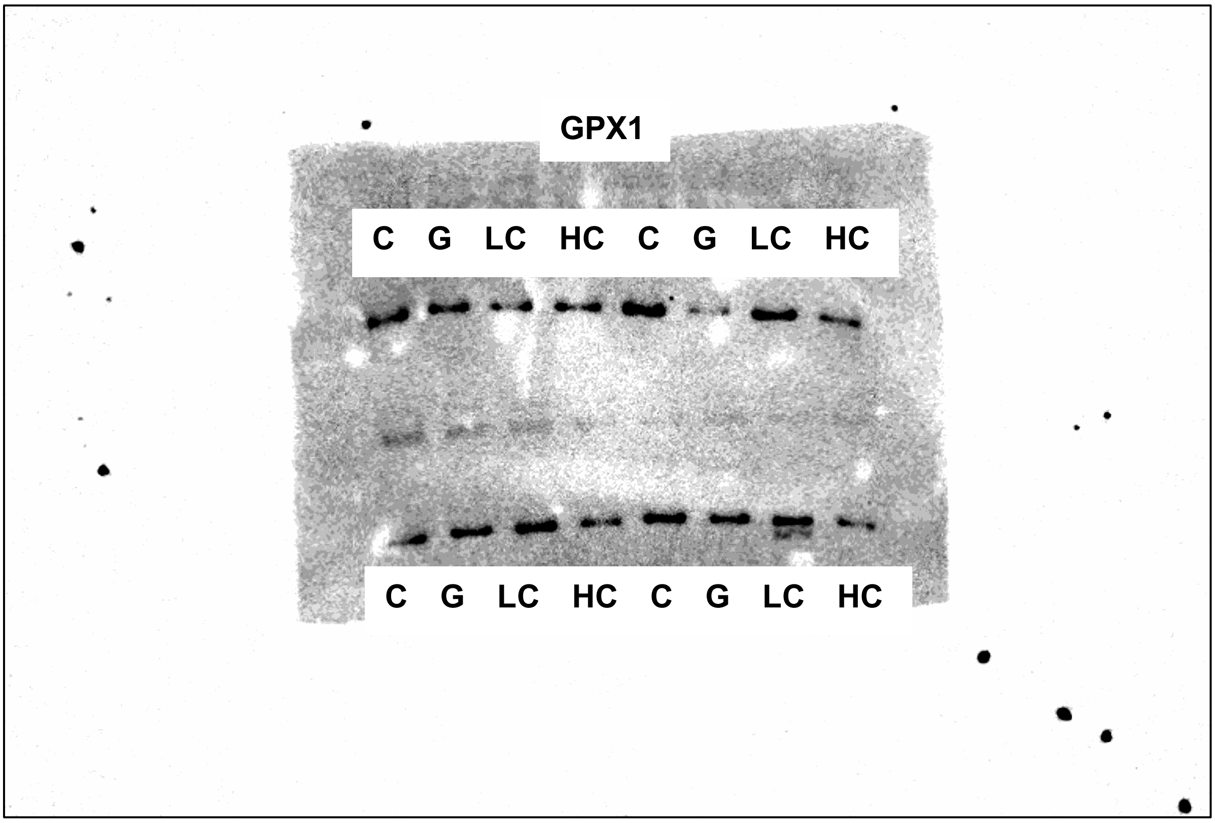
GPX1
C G LC HC C G LC HC
C G LC HC C G LC HC

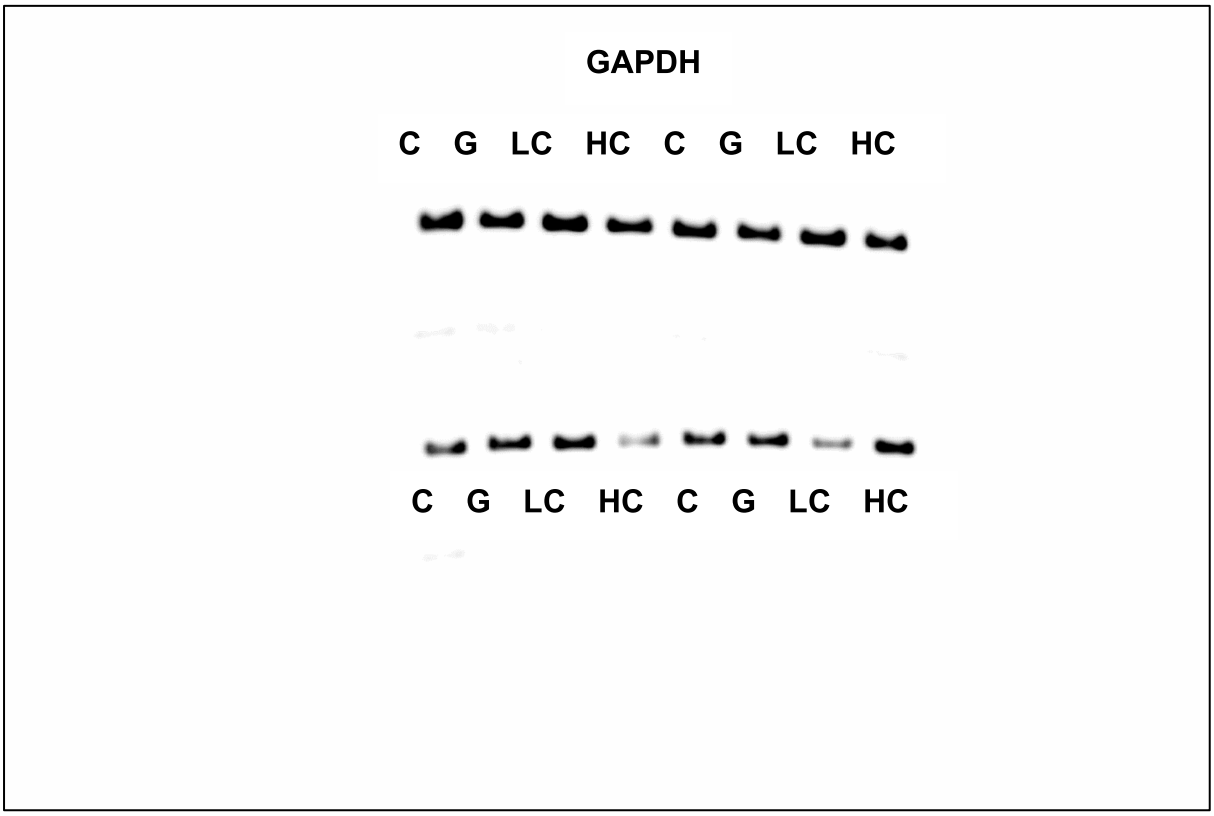
GAPDH
C G LC HC C G LC HC
C G LC HC C G LC HC

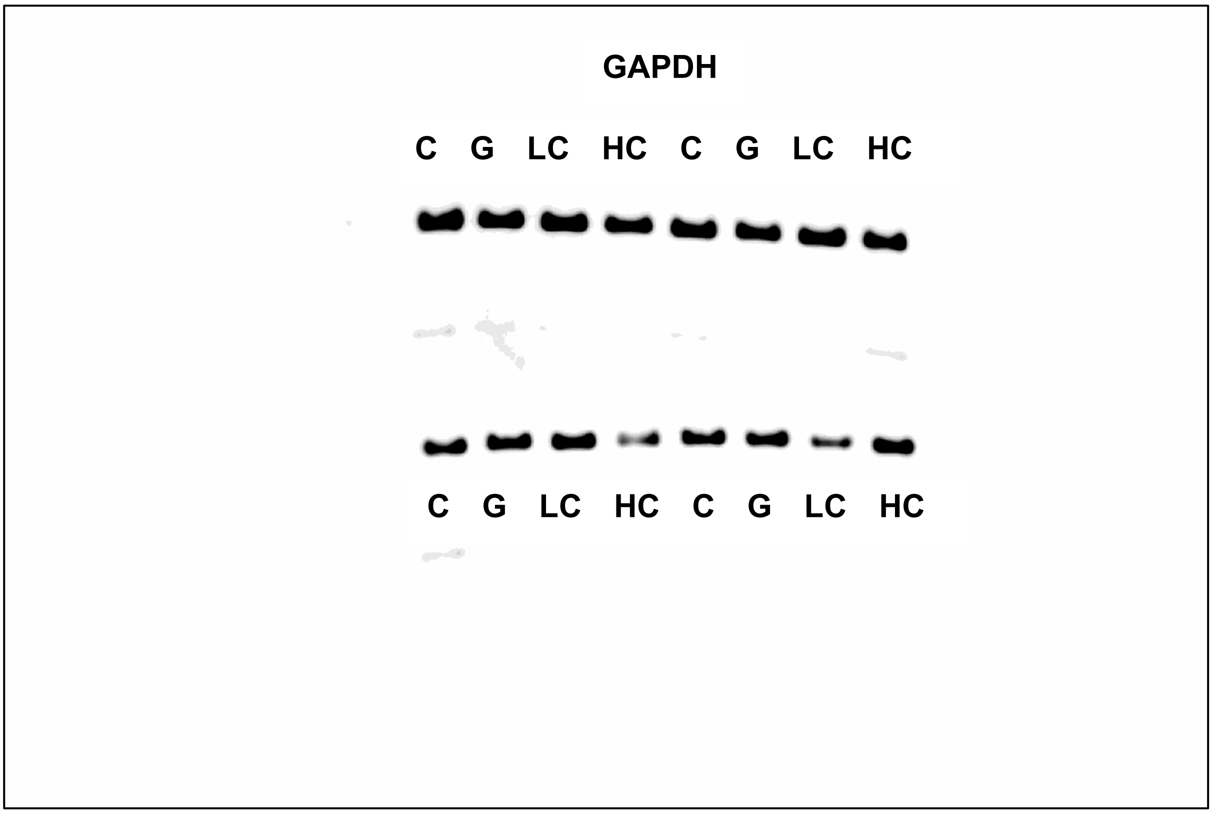
GAPDH
C G LC HC C G LC HC
C G LC HC C G LC HC

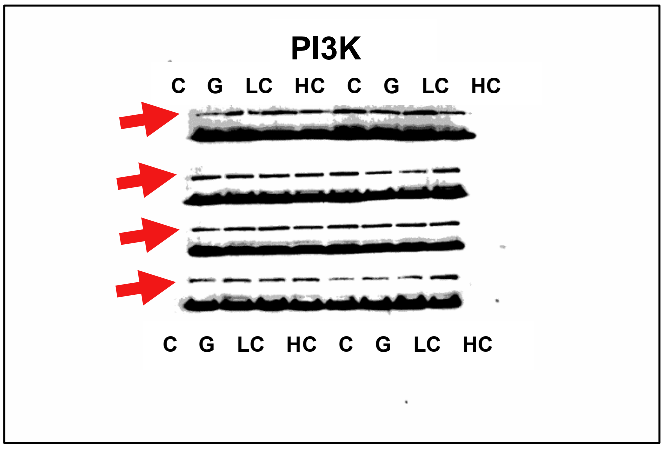
PI3K
C G LC HC C G LC HC
C G LC HC C G LC HC

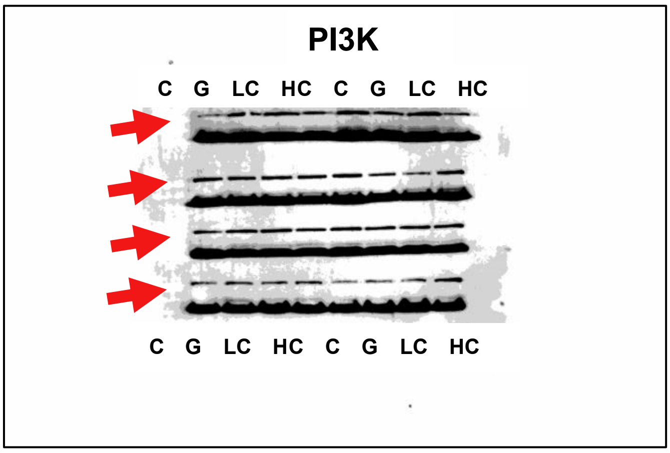
PI3K
C G LC HC C G LC HC
C G LC HC C G LC HC

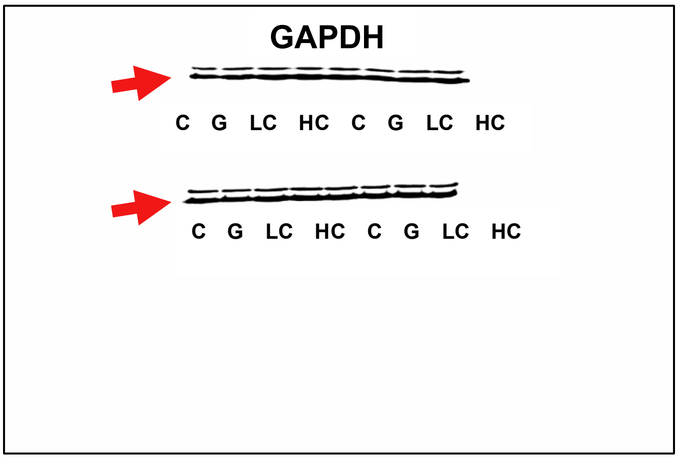
GAPDH
C G LC HC C G LC HC
C G LC HC C G LC HC

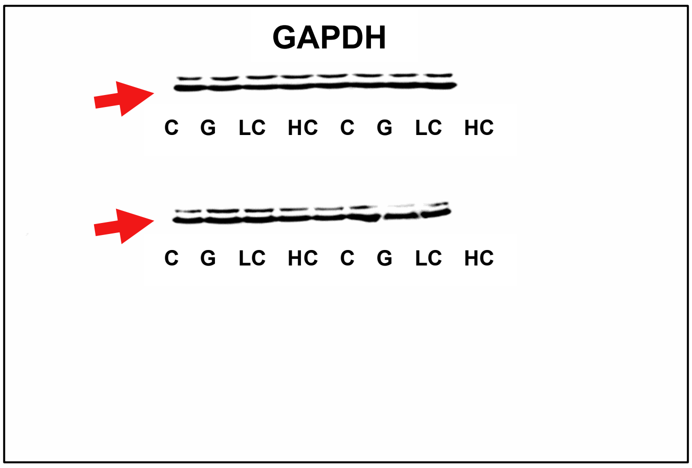
GAPDH
C G LC HC C G LC HC
C G LC HC C G LC HC

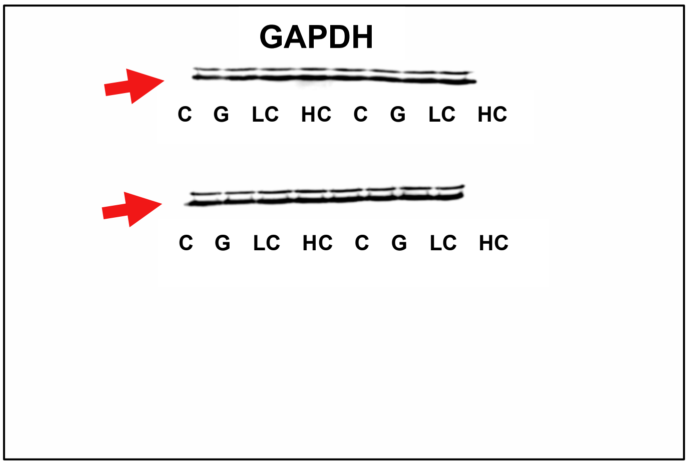
GAPDH
C G LC HC C G LC HC
C G LC HC C G LC HC

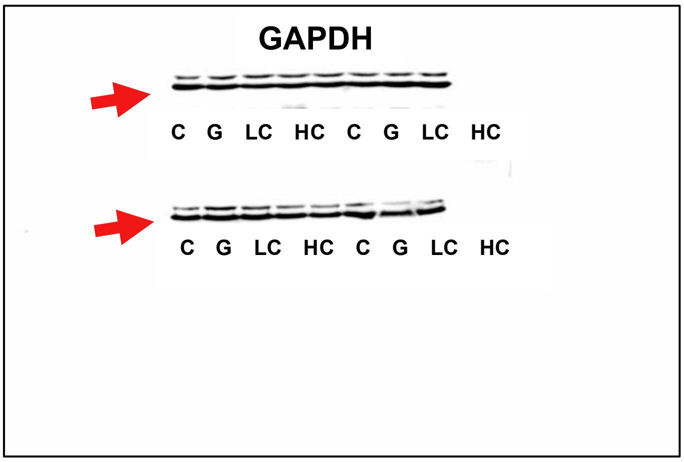
GAPDH
C G LC HC C G LC HC
C G LC HC C G LC HC

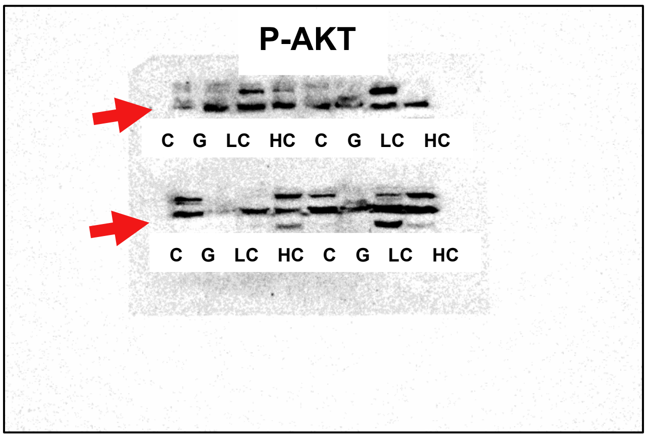
P-AKT
C G LC HC C G LC HC
C G LC HC C G LC HC

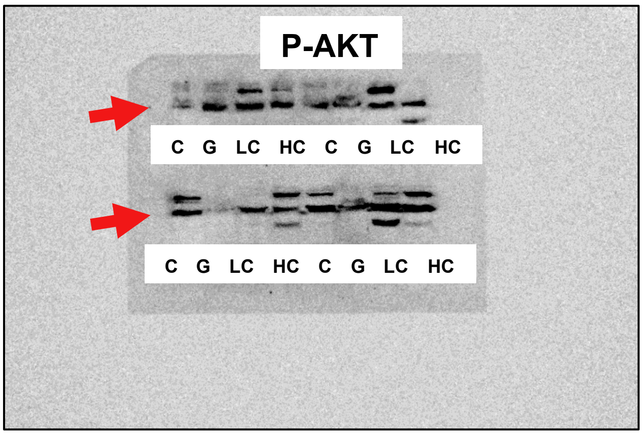
P-AKT
C G LC HC C G LC HC
C G LC HC C G LC HC

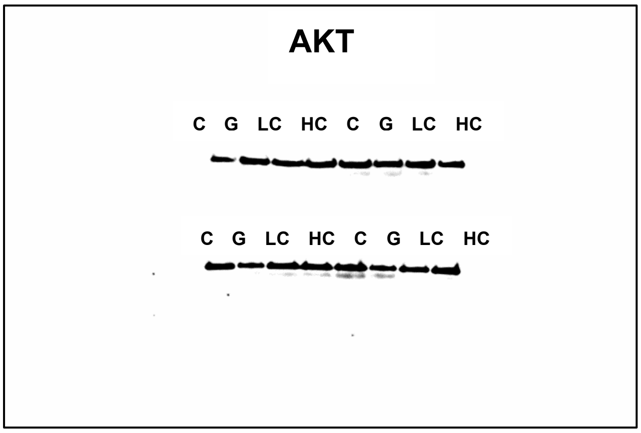
AKT
C G LC HC C G LC HC
C G LC HC C G LC HC

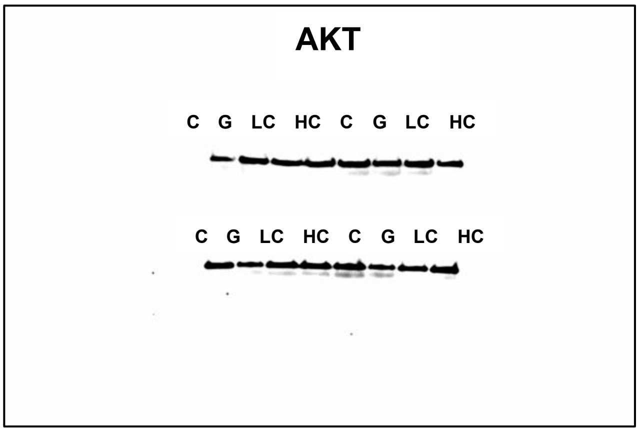
AKT
C G LC HC C G LC HC
C G LC HC C G LC HC

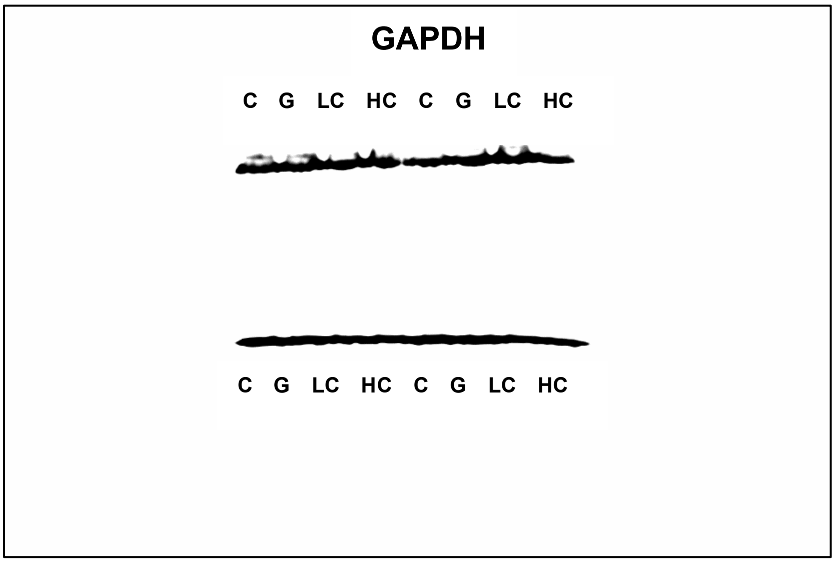
GAPDH
C G LC HC C G LC HC
C G LC HC C G LC HC

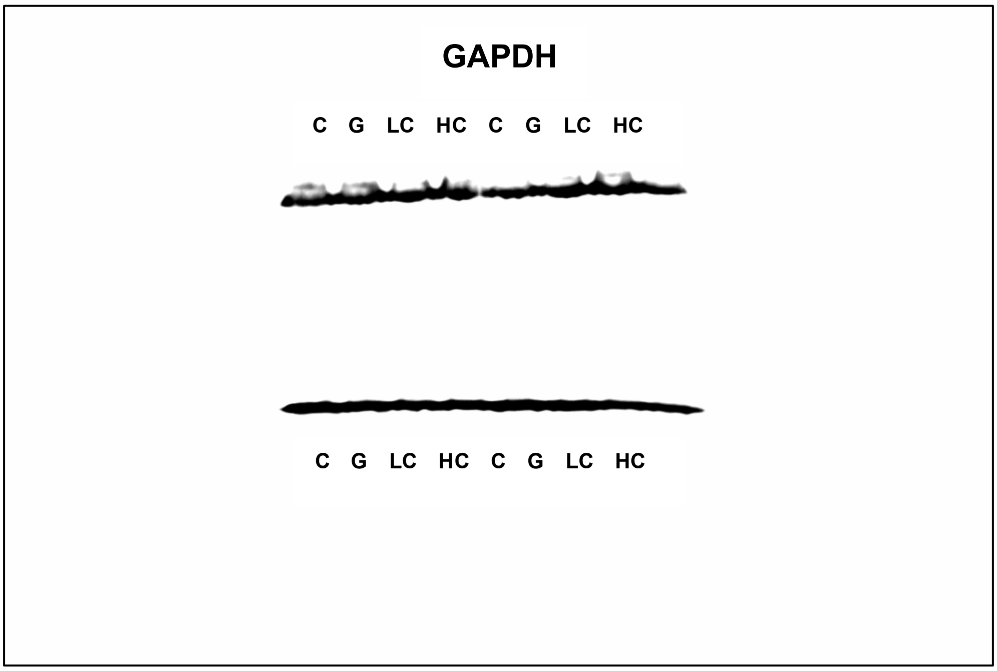
GAPDH
C G LC HC C G LC HC
C G LC HC C G LC HC

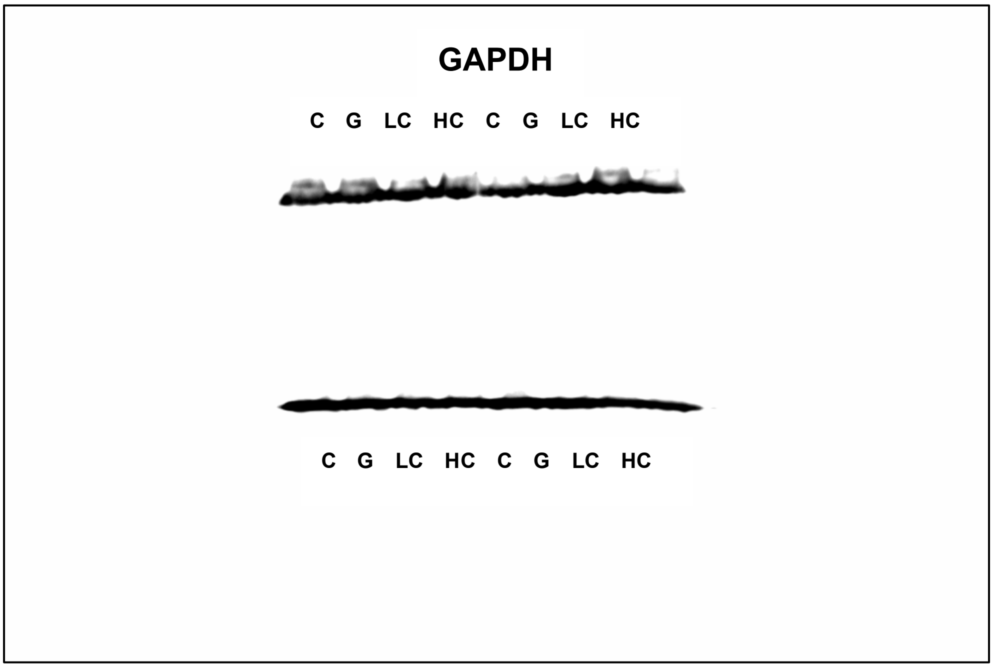
GAPDH
C G LC HC C G LC HC
C G LC HC C G LC HC

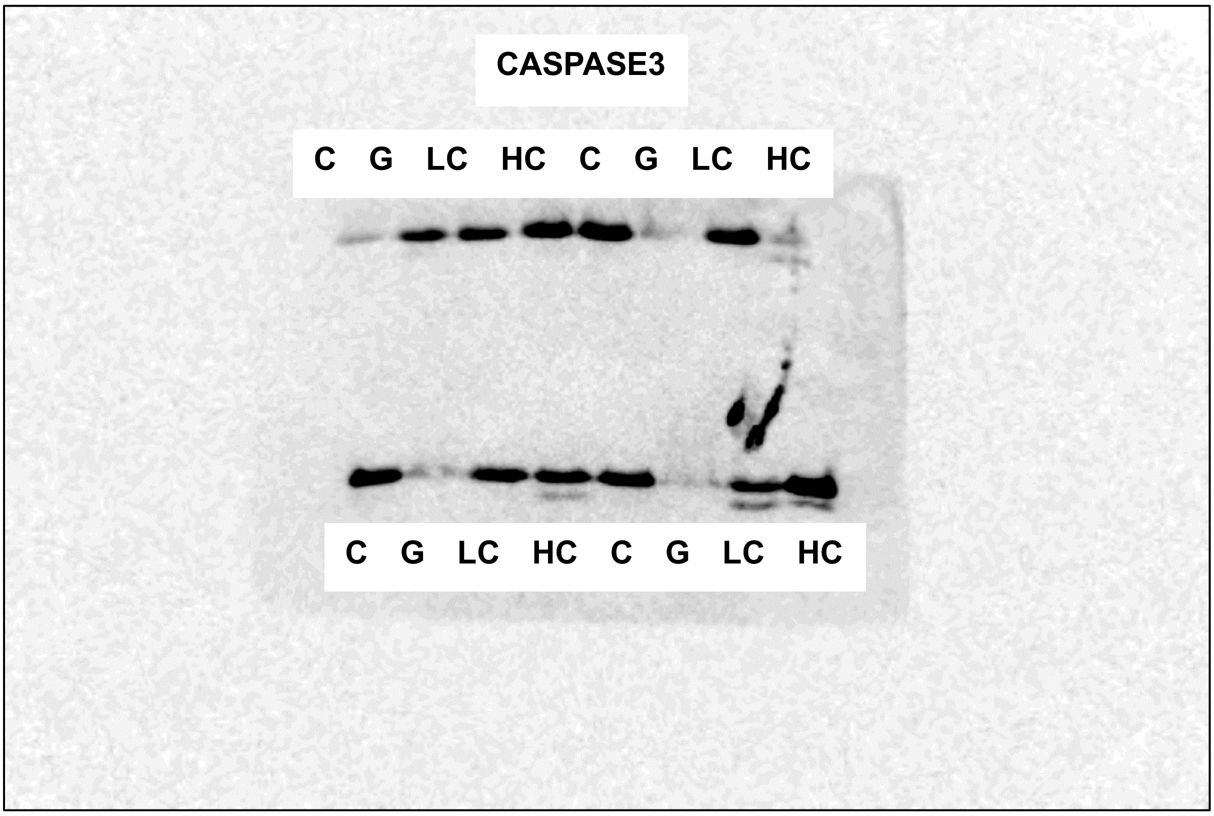
CASPASE3
C G LC HC C G LC HC
C G LC HC C G LC HC

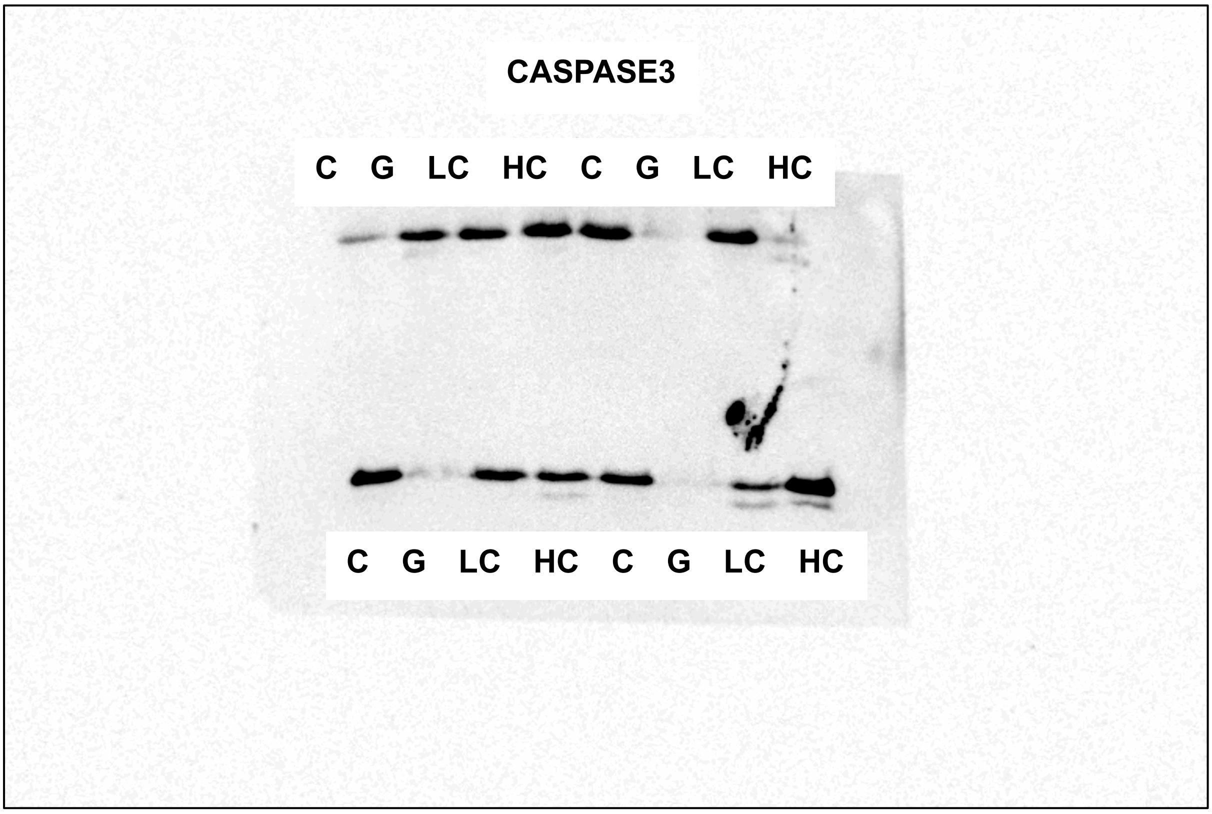
CASPASE3
C G LC HC C G LC HC
C G LC HC C G LC HC

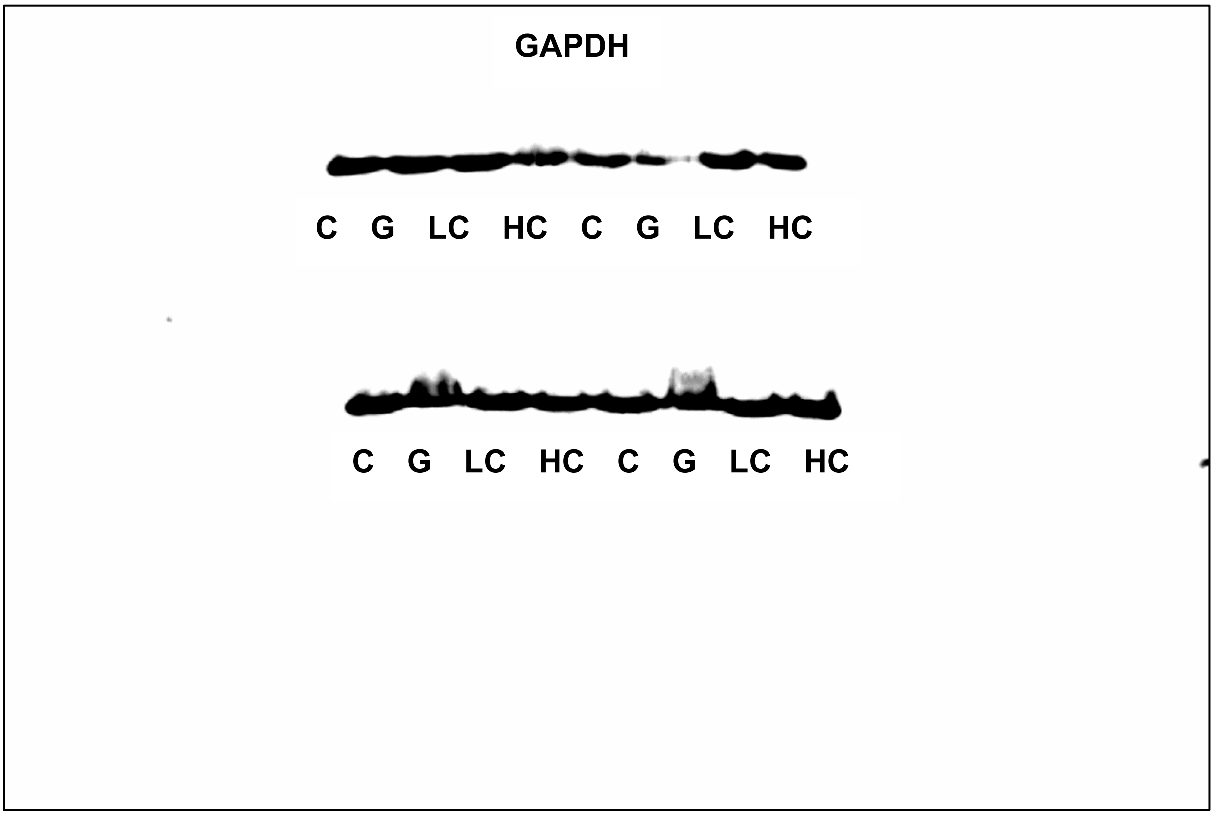
GAPDH
C G LC HC C G LC HC
C G LC HC C G LC HC

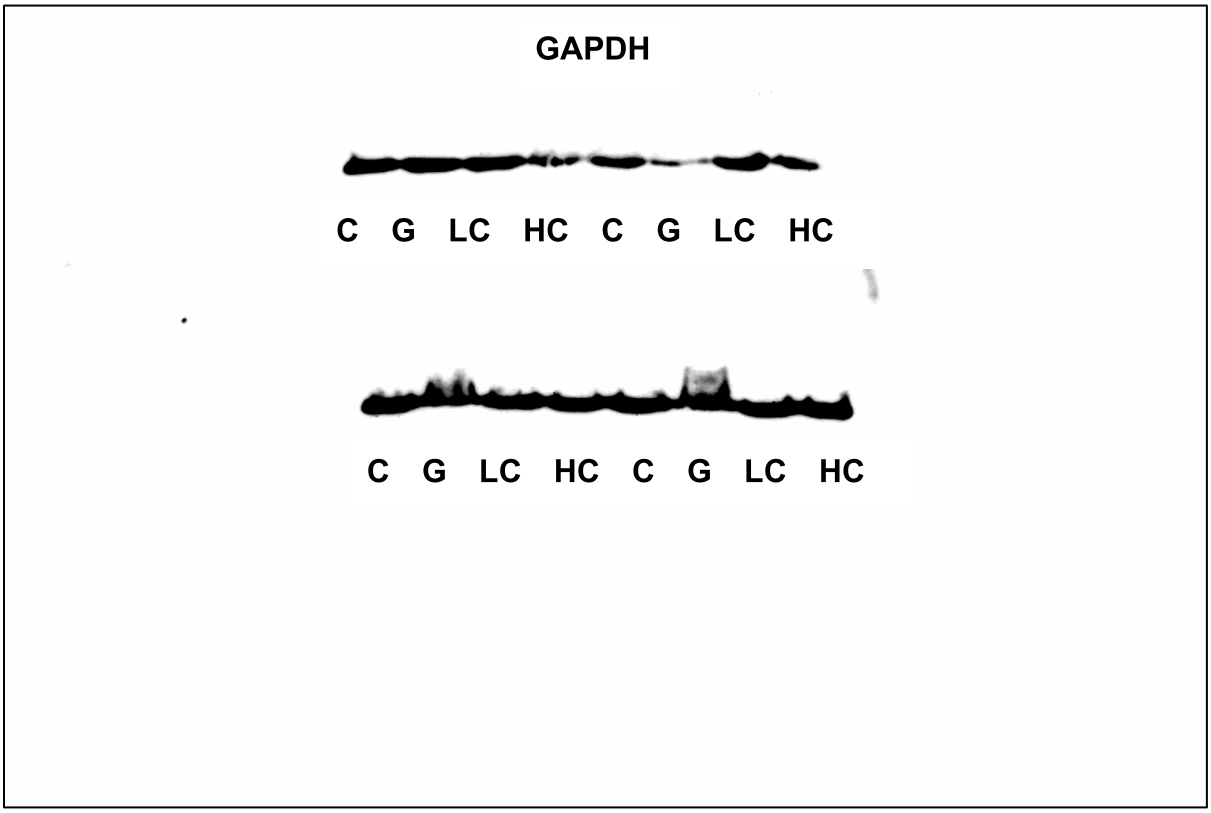
GAPDH
C G LC HC C G LC HC
C G LC HC C G LC HC
