# Supplementary material for: Cacao powder supplementation attenuates oxidative stress, cholinergic impairment, and apoptosis in d-galactose-induced aging rat brain
Source: Sci Rep. 2021 Sep 9;11:17914. doi: 10.1038/s41598-021-96800-y (PMC8429651; doi:10.1038/s41598-021-96800-y)

Cacao powder supplementation attenuates oxidative stress, cholinergic impairment, and apoptosis in D-galactose-induced aging rat brain

Hyoeun Yoo and Hyun-Sook Kim

Supplementary Table S1. Composition of cacao powder.

Component	Unit	Cacao powder (g/100g)
Carbohydrate	g/100g	52.0
Protein	g/100g	26.5
Lipid	g/100g	11.8
Saturated Fatty acid	g/100g	6.87
Monounsaturated Fatty acid	g/100g	3.62
Polyunsaturated Fatty acid	g/100g	0.432
Fiber	g/100g	27.2
Water	g/100g	3.95
Total Flavonoids	mg/g	0.411

Supplementary Table S2. Composition of the experimental diets.

Composition	Normal Diet (for C)	Normal Diet (for G)	10% Cacao (for LC)	16% Cacao (for HC)
**Macronutrient composition**				
**Carbohydrate, % of energy**	75.92	75.92	75.92	75.92
**Protein, % of energy**	14.70	14.70	14.70	14.70
**Fat, % of energy**	9.35	9.35	9.35	9.35
**Energy, kcal/kg**	3850	3850	3921.80	3964.92
**Nutrient Composition (g/kg)**				
**Casein**	140.00	140.00	112.41	95.86
**Sucrose**	100.00	100.00	100.00	100.00
**Maltodextrin**	125.00	125.00	125.00	125.00
**Corn Starch**	495.692	495.692	463.892	444.804
**Cellulose**	50.00	50.00	21.68	4.69
**Soybean Oil**	40.00	40.00	27.71	20.34
**Crude protein**	141.80	141.80	141.80	141.80
**Crude lipid**	40.00	40.00	40.00	40.00
**Crude fiber**	50.00	50.00	50.00	50.00
**Tertiary butylhydroquinone (TBHQ)**	0.008	0.008	0.008	0.008
**Mineral mix**	35	35	35	35
**Vitamin mix**	10	10	10	10

**L-Cystine**	1.8	1.8	1.8	1.8
**Choline Bitartrate**	2.5	2.5	2.5	2.5
**cacao powder**	0	0	100.00	160.00
**Total**	1000.0	1000.0	1000.0	1000.0

Supplementary Figure S1. Effect of cacao on protein expression levels of antioxidant enzymes.

(a) Western blot figures of SOD1, GAPDH; (b) CAT; (c) GPx1, GADPH.

C, control; G, D-galactose-induced aging; LC, D-galactose injection with 10% cacao powder mixed diet; HC, D-galactose injection with 16% cacao powder mixed diet.

(a) SOD1

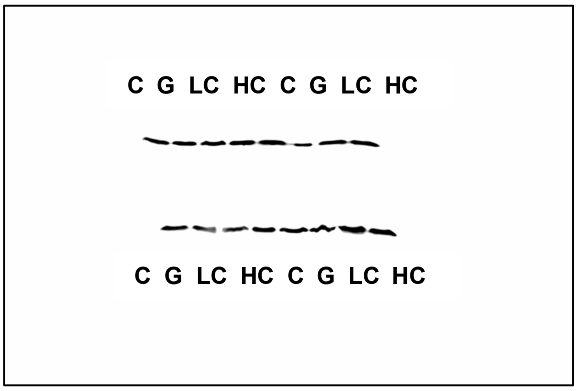

GAPDH

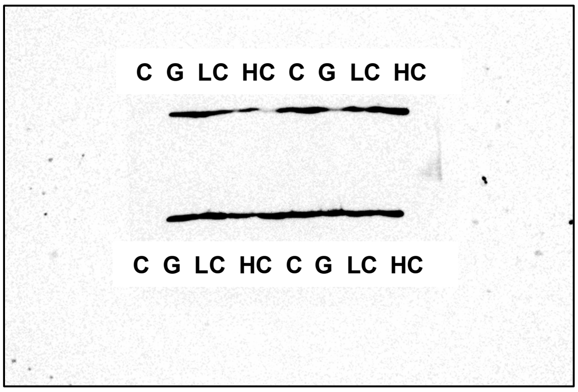

(b) CAT

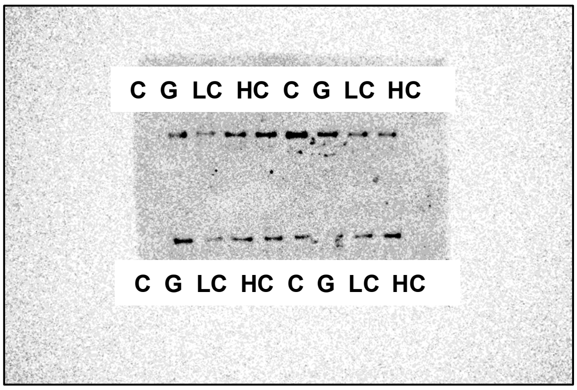

(c) Gpx1 GAPDH

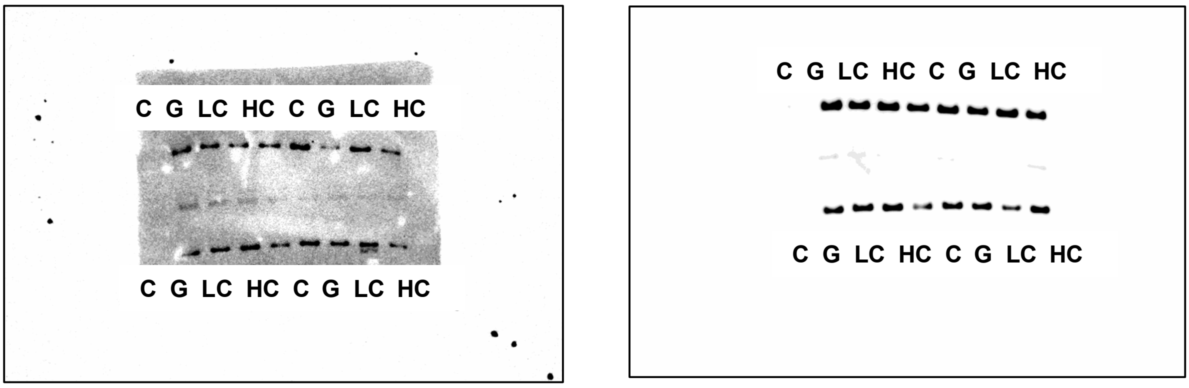

Supplementary Figure S2. Effect of cacao supplementation on protein expression levels of neuroprotective enzymes.

(a) Western blot figures of PI3K; (b) phospho-Akt/Akt; (c) procaspase-3.

C, control; G, D-galactose-induced aging; LC, D-galactose injection with 10% cacao powder mixed diet; HC, D-galactose injection with 16% cacao powder mixed diet.

(a) PI3K

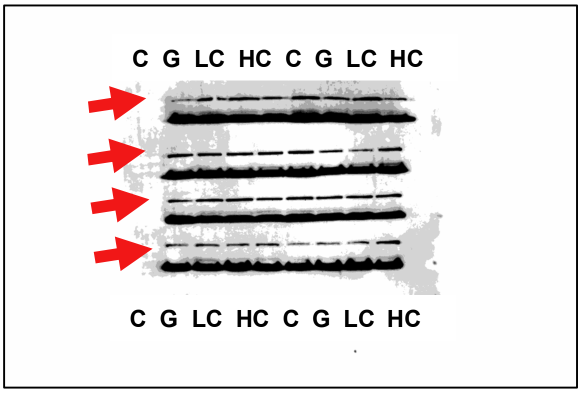

(b) phospho-Akt

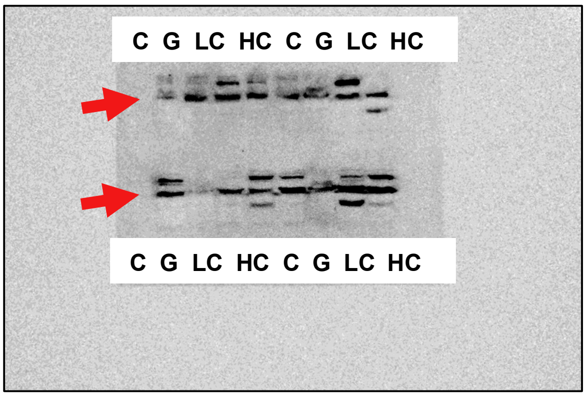

Akt

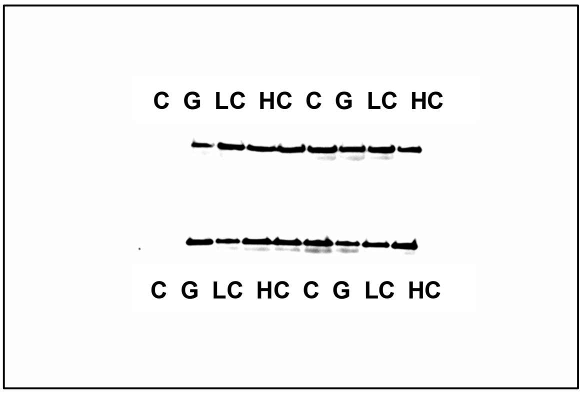

(c) procaspase-3

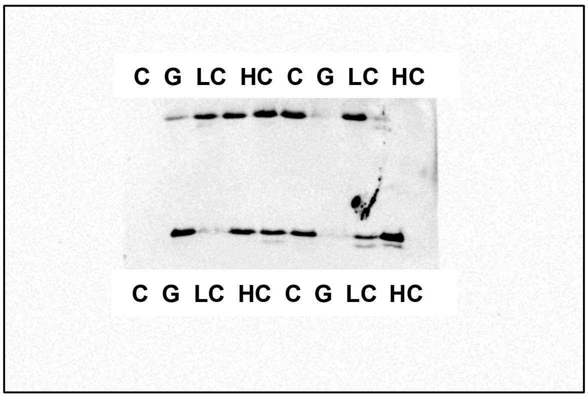

GAPDH

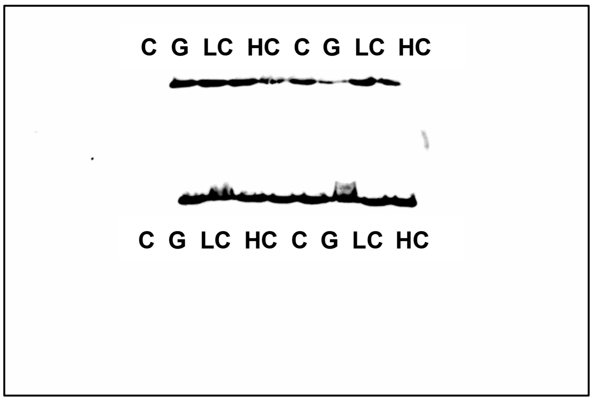

Supplement: Supplementary file 2 — Supplementary Information 2. [file 41598_2021_96800_MOESM2_ESM.pdf]
